# Difference Inadaptive Dispersal Ability Can Promote Species Coexistence in Fluctuating Environments

**DOI:** 10.1371/journal.pone.0055218

**Published:** 2013-02-01

**Authors:** Wei-Ting Lin, Chih-hao Hsieh, Takeshi Miki

**Affiliations:** 1 Institute of Oceanography, National Taiwan University, Taipei, Taiwan; 2 Institute of Ecology and Evolutionary Biology, National Taiwan University, Taipei, Taiwan; McGill University, Canada

## Abstract

Theories and empirical evidence suggest that random dispersal of organisms promotes species coexistence in spatially structured environments. However, directed dispersal, where movement is adjusted with fitness-related cues, is less explored in studies of dispersal-mediated coexistence. Here, we present a metacommunity model of two consumers exhibiting directed dispersal and competing for a single resource. Our results indicated that directed dispersal promotes coexistence through two distinct mechanisms, depending on the adaptiveness of dispersal. Maladaptive directed dispersal may promote coexistence similar to random dispersal. More importantly, directed dispersal is adaptive when dispersers track patches of increased resources in fluctuating environments. Coexistence is promoted under increased adaptive dispersal ability of the inferior competitor relative to the superior competitor. This newly described dispersal-mediated coexistence mechanism is likely favored by natural selection under the trade-off between competitive and adaptive dispersal abilities.

## Introduction

Species dispersal plays important roles in biodiversity patterns observed in nature [1]. Dispersal facilitates coexistence of competing species that otherwise might not coexist [2], [3]. One mechanism of this “dispersal-mediated coexistence” is based on competition-colonization trade-offs [4], [5], [6], [7]. Most models of this mechanism assume that dispersal among patches occurs markedly slower than demographic processes within local patches, including reproduction, competition, and mortality (but see [8], [9], [10], and Discussion), and only considers establishment and extinction of local populations. Coexistence is promoted when the inferior species in within-patch competition exhibits higher dispersal rates and colonizes empty patches more efficiently. However, animal dispersal sometimes occurs at the same timescale as demographic processes [2]. Under these conditions, explicit models describe the demographic processes in local patches, and theory predicts that random dispersal promotes species coexistence via two mechanisms different from competition-colonization trade-offs i.e., “source-sink coexistence” and “emigration-mediated coexistence” [5], [11], [12], [13], [14], [15].

Random, diffusion-like dispersal results in net movement from patches of higher population density to patches of lower population density. When competitive hierarchy of competing species differs among habitat patches, each species has its own suitable patches (where the species is sustained in the absence of dispersal). Random dispersal from suitable to unsuitable patches leads to within-patch coexistence (source-sink coexistence [5], [11], [14]). These predictions have been demonstrated by experimental studies, where local communities are connected by random dispersal (e.g., [16], [17]; see also [18] for meta-analysis). When different competitive hierarchies are not observed among patches i.e. one species is dominant, and all other species are excluded in the absence of dispersal, random dispersal can promote coexistence. Coexistence can occur if the superior competitor increases mobility and exhibits stronger maladaptive net emigration from the patches of higher reproduction rate. Maladaptive dispersal weakens inter-specific competition, and therefore enables the inferior competitor to persist (emigration-mediated coexistence [12], [13], [15], [19], [20]).

However, it is counterintuitive that the superior competitor moves maladaptively if dispersal results from natural selection. In these models, the assumed dispersal randomness, in which movement rate is neither affected by individual conditions nor by environmental variations, could result in maladaptive dispersal. In nature, dispersal may not be as random and maladaptive as assumed by the models. Indeed, some organisms actively determine movement direction and effort using fitness-related cues in order to improve growth, survival, and/or reproduction ([21], [22]). For example, phytophagous insects respond to chemical [23] and optical [24] cues from green leaves. In addition, by selecting habitat and food, mobile crustacean grazers affect the ecosystem differently than passive dispersers [25]. We denote active, fitness-motivated movements as “directed dispersal”, which is likely to result in adaptive net movement.

Directed dispersal has been modeled as density-dependent dispersal, where movement is based on environmental suitability and the density of local population (e.g., [26], [27]). This density-dependent dispersal is proposed as a mechanism to regulate total population size [27] and spatial distribution [26]. In the context of competition, density-dependent dispersal is also used to explain coexistence pattern [28], and it realizes the spatially segregated distribution of populations, which, promotes coexistence of directly-competing species whose competition outcomes are otherwise determined by priority effect [29]. Directed dispersal based on fitness differences between patches are also studied in the single species context, and theoretical studies show that this fitness-dependent dispersal can affect resource distribution [30] and stabilize total population size by creating asynchrony of population sizes and resource levels among patches [31], [32]. Fitness-dependent dispersal is also known to affect species interaction with its predator [33], [34].

It is natural to ask how these population and resource dynamics affect a species’ interaction with its competitor because temporal variation of resource is a critical factor for dispersal to be adaptive [35]. For example, synchrony of resource levels could affect the susceptibility of a metacommunity to the invasion of another species that adopts fitness-dependent dispersal. Focusing on similar fitness-dependent dispersal modes, we address this new ecological question: How does directed dispersal affect coexistence of competing species?

Theoretical studies that address this question remain limited. Armsworth and Roughgarden [36] developed a discrete-time model of Lotka-Volterra competition to compare the biodiversity outcomes of random and fitness-dependent dispersal that is based on neighboring information. They reported that random dispersal increased local diversity and community similarity; however, fitness-dependent dispersal had no effect on either community attributes. Amarasekare [19] developed a three-patch model of one common resource, two competitors, and one top predator. In her model, each of the two competitors dominates one of two patches respectively, and they coexist in the third patch in the absence of dispersal. She found that dispersal mode is critical to dispersal-mediated coexistence, and directed dispersal is maladaptive when contingent on competitor and predator densities. This form of density-dependent dispersal could promote emigration-mediated coexistence consistent with random dispersal [19].

Armsworth and Roughgarden [36] and Amarasekare [19] focused on adaptive fitness-dependent dispersal; however the results indicated the adaptive dispersal did not promote species coexistence. One possible mechanism is that the optimal strategy for directed dispersers is to be sedentary when resources do not fluctuate substantially [35]. However, when consumer-resource interactions result in oscillations in resource abundance and patch qualities a sedentary lifestyle is not an optimalstrategy [37]. Another potential mechanism is that both previous models assumed that competitive hierarchies differed among patches. Consequently, each adaptive dispersing species concentrates on its own suitable patch, which is unsuitable for other species. Therefore, species are spatially segregated by adaptive dispersal. Nevertheless, under conditions where adaptive movement does not segregate species, the role of adaptive movement on competing species remains unclear.

In the present study, we focused on a metacommunity where relative patch suitability is not species-specific; for two competing species, the identity of the better patch is always the same. Models with this setting have been studied by Abrams and Wilson [20] and by Namba and Hashimoto [15] for the cases of random dispersal. Our primary question was under what conditions directed dispersal was adaptive, maladaptive, or had no fitness effects. In addition, in each case (adaptive or maladaptive), how did directed dispersal affect coexistence of competing species.

Our study was based on a two-patch model, comprised of two mobile consumers competing for one sedentary resource species. While previous models [19], [36] focus on the mechanism of local coexistence (at one patch) of species that already coexist in the metacommunity even without dispersal, we focus on the mechanism of regional coexistence (in the two-patch system) by assuming that one superior species dominated both patches andregional coexistence was impossible without dispersal. Thus, in our model, any advantage of the inferior over the superior species for coexistence must be accomplished by dispersal, either dispersal of itself or the superior species. Within this setting, we were able to address the spatial mechanisms of coexistence without confounding with the source-sink process that promotes within-patch coexistence of species that already coexist regionally. We compared the adaptiveness of dispersal and the competitive outcomes of two competing consumers, when the consumers adopted random or directed dispersal. We also compared the results from environments with different stability in resource availability. Under these conditions, we determined a new mechanism of dispersal-mediated coexistence. We demonstrated that directed dispersal was adaptive when species track oscillations in resource availability. Species coexistence was promoted when the inferior competitor exhibited higher movingcapacity than the superior competitor.

## Methods

### Model

#### Metacommunity model: general formulation

The effects of different dispersal modes on the outcome of competition between two competing consumers were investigated using an ordinary differential equation (ODE)-based metacommunity model. We assumed two patches, and each of two local communities was comprised of three species; one resource species, and two consumers competing to exploit the resource species. We also assumed a sedentary resource species; local communities were linked by consumer dispersal between patches. 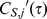 and 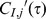 denoted superior (*S*) and inferior (*I*) consumer species population densities, respectively, and *R_j_*(*τ*) represented the resource species population density in patch *j*(*j = *1, 2) at time *τ*. The dispersal function, 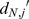 (*N* = *S* or *I*) was a linear function of 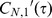 and 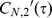, representing the net increase of 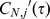 through dispersal (immigration – emigration). The exact dispersal function formulations depended on “dispersal mode”, and are specified later in this section.

The general model formulation was described as follows:
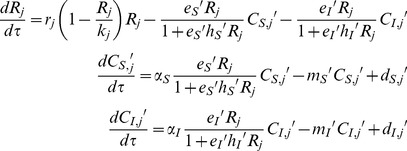
(1)


We assumed consumer-resource interaction based on Hollings Type II functional response, with the resource species exhibiting logistic growth. Here, *r_j_* was the intrinsic growth rate, *k_j_* was the resource species carrying capacity in patch *j*(*j* = 1,2), 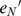 was the encounter rate, 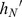 was the handling time, *α_N_* was the assimilation rate, and 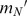 was the per capita mortality rate of the superior (*N* = *S*) or inferior (*N* = *I*) consumer.

For simplicity, we assumed *r_1_* = *r_2_* = *r*, sothat two patches differed only in carrying capacity; and we assumed *α_S_* = *α_I_* = *α*, *m_S_* = *m_I_* = *m*, so that the superior and inferior consumer differed only in foraging ability, which was calculated by encounter rate and handling time. Then, let *t = τ/r*,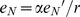, 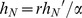, 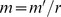, 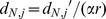, and 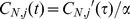.We rescaled the model as follows:
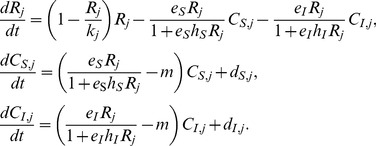
(2)


The two consumer species were “competing consumers”, which competed by exploiting and subsequently depleting the common resource. An isolated patch supported only a minimal resource level R*_S, min_ and R*_I, min_ for the superior and inferior consumer to maintain their respective population at a steady state. Generally, the inferior competitor showed a higher R*_min_ value and was excluded in the absence of dispersal [38]. We assumed the consumer species had the potential to maintain its population in either patch in the absence of dispersal (i.e., *k*_1_,*k*_2_> R*_I, min_). The inferior species could still persist, and even exclude the superior species without dispersal if the carrying capacity exceeded certain level [39], [40]. Therefore, we chose carrying capacities (*k*_1_, *k*_2_) small enough to cause competition exclusion of the inferior by the superior consumer and then assessed spatial coexistence under two patch conditions.

#### Dispersal modes

In this model, there was no direct cost of dispersal; therefore all emigrants from one patch immediately became immigrants of the other patch. Per capita emigration rate was the product of moving capacity (i.e., maximum potential emigration rate, *d*_max, *N*_) and emigration tendency, which was determined by dispersal mode. The dependence of competitive outcomes of the two competing consumers on the following three dispersal modes was investigated: random dispersal, fitness-dependent dispersal (based on global information), and growth-dependent dispersal (based on local information).

In the random dispersal mode, the emigration tendency for random dispersal was maintained at the maximum level (* = *1.0) in both patches. Subsequently, the dispersal function of consumer *N* (*N* = *S* for the superior or *N* = *I* for the inferior) in patch 1was given by:
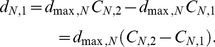
(3)


Subsequently, the dispersal function of consumer *N* in patch 2 was*–d_N,_*_1_.

With random dispersal, our model is the same as Namba and Hashimoto’s model [15], and accordingly is expected to allow coexistence through the emigration-mediated process.

In the fitness-dependent dispersal mode, which is based on global information, we assumed organisms have immediate information regarding fitness (i.e., the per capita growth rate)in both patches under fitness-dependent dispersal. Therefore, individuals did not emigrate from the patch of higher fitness. Fitness was denoted as *f_N,j_*, and from eq. 2,
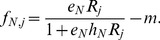
(4)


The emigration tendency from the low-fitness patch smoothly increased with the difference in fitness. The dispersal function of consumer *N* (*N* = *S* for the superior or *N* = *I* for the inferior) in patch 1was:
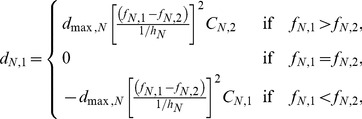
(5)


Then, the dispersal function of consumer *N* in patch 2 was*–d_N,_*_1_. The maximum fitness difference was 1*/h_N_*, which was reached when one patch had an infinite resource level and the other patch had no resource. We used quadratic function for fitness-dependent dispersal to avoid the abrupt change in net dispersal rate when the identity of the patch of higher fitness changes.

In the growth-dependent dispersal mode, which is based on local information, under growth-dependent dispersal, we assumed that consumers had no information concerning the other patch. Emigration tendency depended on the current growth rate, relaxing the strong assumption of global information in the fitness-dependent dispersal mode. If the resource level in the current patch was infinite, the growth rate reached its maximum, 1*/h_N_*. Emigration tendency increased in proportion to the difference between maximum and current growth rate, scaled to maximum growth rate, as follows:
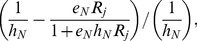
(6)for consumer *N* from patch *j*. Therefore, the dispersal function of consumer *N* in patch 1, *d_N,_*_1_, was



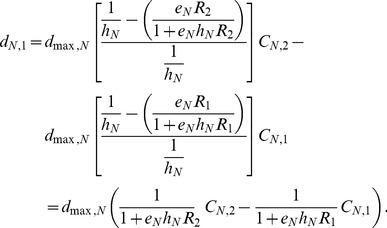
(7)The dispersal function of consumer *N* in patch 2 was *–d_N,_*_1_.

### Analysis

Model analyses included mathematical and numerical approaches. For the two directed dispersal modes (for fitness-dependent dispersal, see Text S1;for growth-dependent dispersal, see Text S2), we mathematically analyzed the coexistence criteria under two limiting conditions: (1) both consumers moved rapidly, and (2) one species moved rapidly, while the other was sedentary. Since it is impossible to exploit all parameter space by numerical simulations only, the mathematical approach helps us to obtain robust results independent of parameter values in specific situations (e.g. when the dispersal ability is very high).

Our major results were based on numerical analyses for three dispersal modes. We assumed the two consumers adopted the common dispersal mode, and we explored the effects of a rescaled moving capacity (*d*_max,*N*_) for both species. We also considered three environments that differed in carrying capacities (*k*_1_and*k*_2_) for resource species. The three environments produced different dynamics between the superior consumer (*C_S_*) and the resource (*R*) when isolated, and in the absence of the competitor: (1) under low carrying capacity conditions, *C_S_*-*R* dynamics reached a stable equilibrium in both patches (*k*_1_ = 0.6, *k*_2_ = 0.3); (2) under intermediate carrying capacity conditions, C*_S_*-*R* dynamics reached a stable equilibrium in one patch (*k*_2_ = 0.4), but displayed periodic oscillations in the other (*k*_1_ = 0.8) patch; and (3) under high carrying capacity conditions, C*_S_*-*R* dynamics displayed periodic oscillations in both patches (*k*_1_ = 1.4, *k*_2_ = 0.7). For convenience, we denoted these settings environment 1, environment 2, and environment 3, respectively.

**Figure 1:**
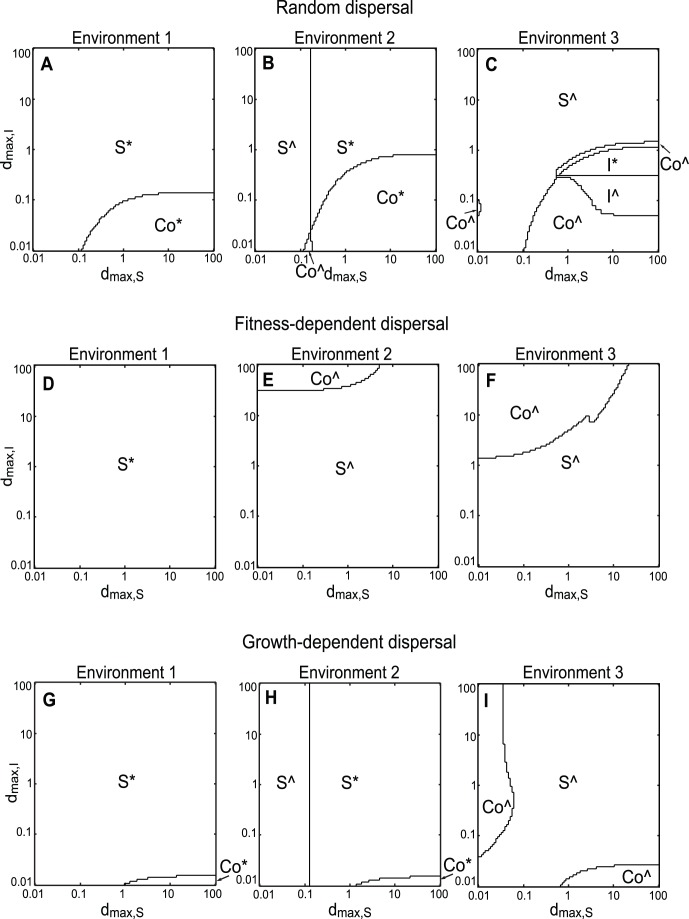
Competition outcomes between two consumer species, depending on moving capacity. In each panel, the species compositions are denoted as follows: S: superior species dominance, Co: coexistence, and I: inferior species dominance. Stable steady states are indicated by (*), and unstable, periodic, or fluctuation outcomes are indicated by (^∧^). Parameters are (*k*_1_, *k*_2_) = (0.6, 0.3) for Environment 1 in (A, D, G), (0.8, 0.4) for Environment 2 in (B, E, H), and (1.4, 0.7) for Environment 3 in (C, F, I).

For simulations, the inferior competitor was the invader, invading an environment occupied by the superior competitor (C*_S_-R* system). We numerically simulated this invasion process using C language, and evaluated the long-term competition outcome in terms of population stability (stable or with fluctuation) and species persistence (see Text S3 for detailed numerical method).We chose the default parameter set based on the parameter set used in [15].Then, we adjusted the value of *h_I_* and *e_I_* so that all possible competitive outcomes we have found in mathematical analysis are included in the parameter regions shown in Figure 1. Specifically, mathematical analysis (Text S2) indicated that with growth-dependent dispersal, coexistence is possible when the inferior has very low and the superior has very high moving capacity if the ratio of minimal resource levels (R*_I_, _min_/R*_S_, _min_) is smaller than 2*k*_2_/(*k*_1_+*k*_2_). Throughout our simulations, we assumed a default parameter set at *h_S_* = 3, *h_I_* = 2.7, *e_S_* = 1, *e_I_* = 0.8, and *m* = 0.1.

To interpret the competition outcomes, we needed to judge the adaptiveness of dispersal. We regarded a dispersal strategy as adaptive if an individual adopting this strategy exhibited increased fitness relative to its sedentary counterpart. In particular, fitness was evaluated by invader fitness, which is theoretically defined as long-term exponential invader growth [41]. In our simulation, the inferior competitor was introduced as an invader to the C*_S_-R* system. Therefore, we evaluated fitness of the inferior by calculating its average growth rate when it starts to invade the C*_S_-R* system. In order to simulate invasion stage long enough to obtain the proper average value while avoiding the effect of invader on the resource, we use a model where the inferior has no effect on the resource. We first evaluated the invader fitness of the sedentary inferior (*d*_max,*I = *_0) to a habitat dominated by the superior with each *d_max, S_* for each environment and dispersal mode. We then calculated the adaptiveness as the difference between the invader fitness of each dispersing population of the inferior and that of their sedentary counterpart. We present only the adaptiveness evaluated based on the invader fitness of the inferior because it corresponds to the scenario of our numerical simulation.

We also calculated the fitness differential that the dispersers experienced when they change patches (see Figure S1). This value may not work as an adaptiveness measure because in the non-equilibrium cases, fitness at one patch may change after dispersers arrives that patch (see Text S4 for more detailed information).

## Results

**Figure 2:**
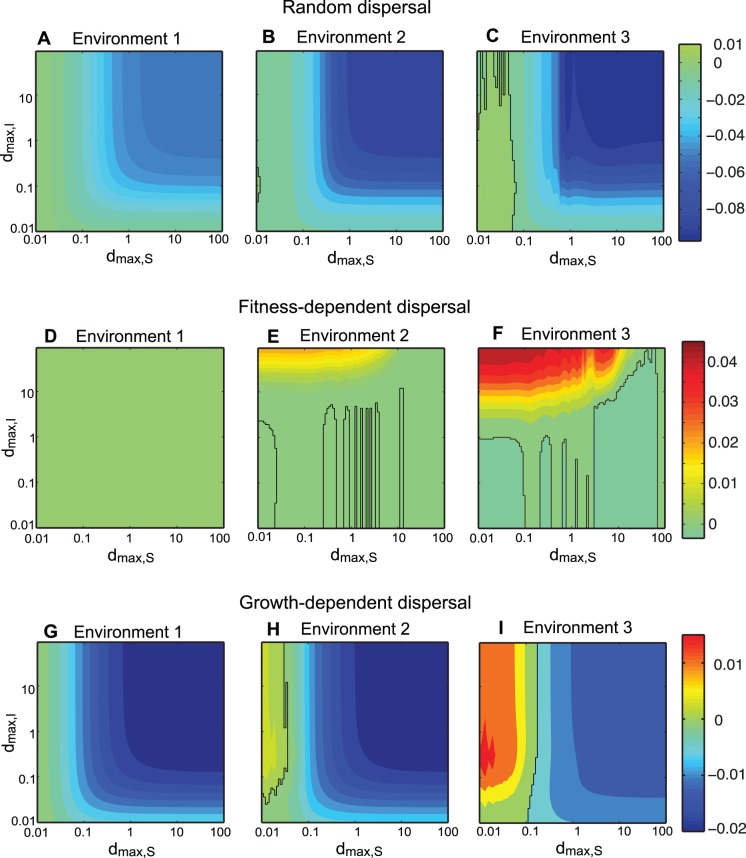
Adaptiveness of dispersal. Adaptiveness was calculated as the difference between the invader fitness of each dispersing population of the inferior and that of their sedentary counterpart. Boundaries of positive and negative values are marked by lines. Note that the color scales are different for different dispersal modes. Parameters are (*k*_1_, *k*_2_) = (0.6, 0.3) for Environment 1 in (A, D, G), (0.8, 0.4) for Environment 2 in (B, E, H), and (1.4, 0.7) for Environment 3 in (C, F, I).

Distinct competitive outcomes of the two consumers emerged, depending on moving capacity of species (*d_max, S_* and *d_max, I_*) (Figure 1). More importantly, different dispersal mode combinations (random, fitness-dependent, or growth-dependent) and environmental stability (low, intermediate, or high carrying capacities) resulted in different relationships between species moving capacity and competitive outcomes (Figure 1).The adaptiveness, calculated as the difference between invader fitness of the dispersing and sedentary population of the inferior, is presented in Figure 2. We could then classify competitive outcome patterns and explained coexistence mechanisms based on the adaptiveness of dispersal.

### Random Dispersal

Random dispersal facilitateda steady-state coexistence when moving capacitywas high for the superior and low for the inferior consumer in environment 1 (low carrying capacity; region Co* in Figure 1A) and in environment 2 (intermediate carrying capacity; Figure 1B). When the superior consumer’s moving capacity was high, random dispersal was maladaptive to the inferior consumer (Figure 2A, B). Coexistence was observed when maladaptive movement of the superior species exceeded the inferior species (Figure 1A,B).

Random dispersal demonstrated competing consumers coexistence with fluctuating populations (region Co^∧^ in Figure 1C) in environment 3 (under high carrying capacities). Generally, coexistence was realized when the moving capacity of the superior was higher relative to the inferior. In cases of high superior dispersal capacity, that random dispersal was maladaptive (Figure 2C). Some complex outcomes were also observed in this environment. Competitive exclusion of the superior species was possible in this scenario because resource was depleted in patch 2, making the environment very unfavorable to the superior that performs fast, random dispersal. Coexistence was also realized when both consumers exhibited poor moving capacity (Figure 1C); this happened when the inferior had higher ratio of its population in the patch with higher resource level compared to the superior. In a limited parameter region between S^∧^ and I^∧^ regions, we also found alternative steady states (i.e. the coexistence with fluctuating population dynamics was also possible, depending on initial conditions), as is shown in [15]. However, we present only the results from our invasion analysis in Figure 1C. Details in these complex outcomes are provided in the supplementary materials (Text S5 and Figure S2, S3, S4, S5).

### Fitness-dependent Dispersal

Coexistence at steady states could not be reached under fitness-dependent dispersal. If the superior competitor and resource attained a stable equilibrium, invasion by the inferior competitor did not happen. Mathematical analyses indicated that only one possible stable steady state outcome exists in the C*_S_-R* system (model without the inferior consumer). This occurs whentwo patches were maintained equally at the minimal resource level of the superior competitor (*R*_1_ * = R_2_ * = R*_S,__min_) (see Text S1). By definition, R*_S,__min_<R*_I,__min_, and the inferior could not invade this steady state (*R*_1_ * = R_2_ * = R*_S,__min_). Simulation outcomes confirmed this mathematical argument; in environment 1, the superior consumer and resource reached stable equilibria, and coexistence was prevented (Figure 1D).

However, results demonstrated coexistence under fitness-dependent dispersal with periodic fluctuations in environment 2 (intermediate carrying capacities) and in environment 3 (high carrying capacities). Coexistence occurred with low moving capacity for the superior and high moving capacity for the inferior consumers (region Co^∧^ in Figure 1E, F). In the cases where superior consumer moving capacity was low (Figure 2E, F), fitness-dependent dispersal was adaptive if moving capacity of the inferior was high.

The above results indicated that fluctuations in resource level (resulting from the consumer-resource interaction with increased carrying capacity) were critical to adaptiveness of fitness-dependent dispersal, and that resource dynamics traits, to some extent, influenced adaptiveness of fitness-dependent dispersal. In order to elucidate these traits, we proposed an index of the “potential fitness-dependent dispersal advantage” relative to the absence of dispersal. We considered an invading consumer (a population so small that its own effect on fitness was negligible), which adopted fitness-dependent dispersal and possessed high mobility, enabling the population to always disperse and concentrate in them ostresource-rich patch. This “ideal” invader enjoyed an “ideal resource level” denoted as *R_ideal_*(*t*), *R_ideal_*(*t*)* = *max(*R*_1_(*t*),*R*_2_(*t*)). Subsequently, the ideal invader was provided an average resource level of 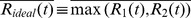. In contrast, a sedentary invader was relegated to one patch, and at best the higher average resource level available was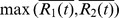. Therefore, the difference between average resource level enjoyed by ideal and sedentary invaders was viewed as an index for the potential advantage of fitness-dependent dispersal; i.e.,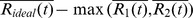. We calculated this index by using *R*_1_(*t*) and *R*_2_(*t*) that are determined by the resident population (the superior consumer), since we needed to consider the situation when a few individuals of the inferior invade a habitat dominated by the superior. If a resource remained more abundant in one patch, the optimal consumer strategy was to stay in the resource-rich patch. Under these conditions, the ideal resource level was equal to the specific patch resource level, and the potential advantage equaled zero. Alternatively, if the resource was sometimes more abundant in one patch and sometimes more abundant in the other, the potential fitness-dependent dispersal advantage exceeded zero. Therefore, fitness-dependent dispersal could be adaptive if moving capacity is high enough (Figure 2E, F), which allows population to reach the patch with high resource levels earlier than the resource levels start to decline there.

**Figure 3:**
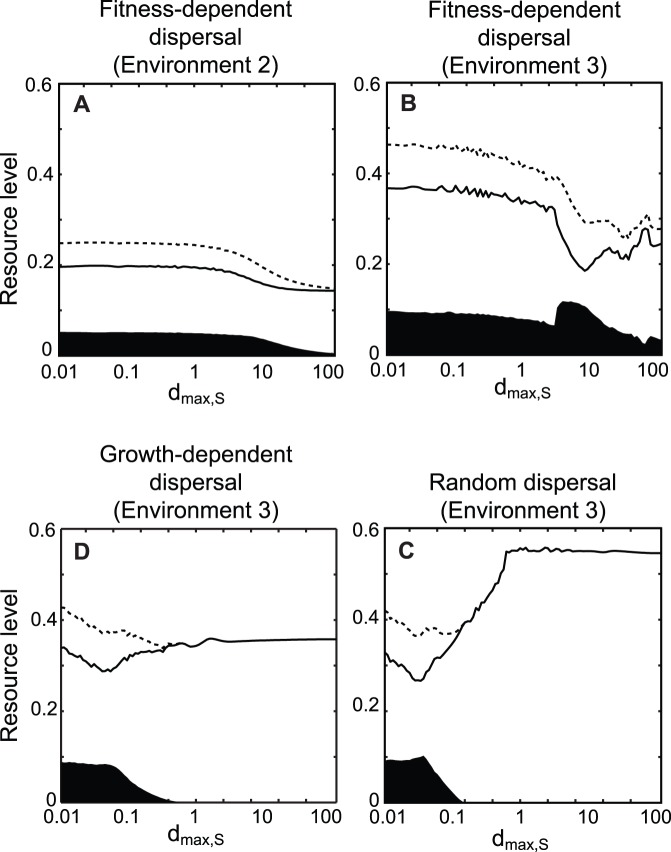
The effect of the superior consumer moving capacity (dmax,S) on resource dynamics. The effect of the superior consumer moving capacity (dmax,S) on resource dynamics when the inferior consumer is absent. These are the resource dynamics that the inferior will face as they invade the community where only the superior resides. Each panel shows the average resource level in patch 1 (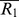, bold lines; for all four cases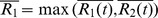), average ideal resource level (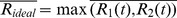, dotted lines), and the potential advantage of fitness-dependent dispersal (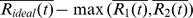, shaded).

**Figure 4:**
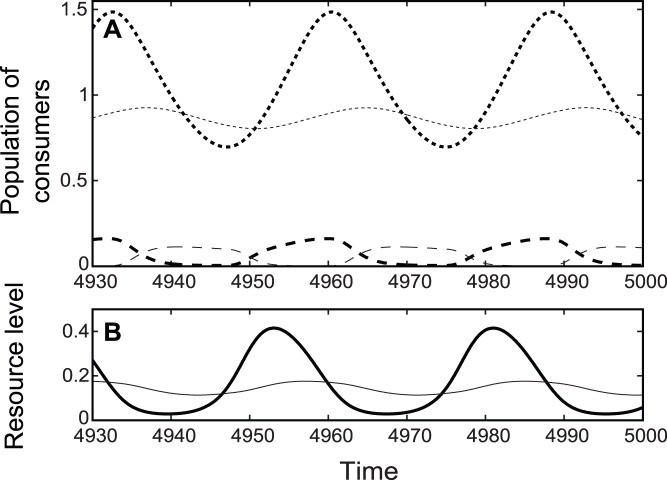
Population dynamics in environment 2 under fitness-dependent dispersal. Population dynamics of consumers (the upper panel, A) and the resource (the lower panel, B) in environment 2 (*k*_1_ = 0.8, *k*_2_ = 0.4) under fitness-dependent dispersal. (Bold solid line: *R*_1_;thin solid line: *R*_2_; bold dotted line: *C_S_*_,1_;thin dotted line: *C_S_*_,2_;bold dashed line: *C_I_*_,1_; thin dashed line: *C_I_*_,2_). The superior consumer moving capacity is low (*d*_max,*S*_ = 0.01), and the inferior consumer moving capacity is high (*d*_max,*I*_ = 100).

The potential advantage of the inferior consumer adopting fitness-dependent dispersal was positive (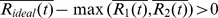) when moving capacity of the superior was low in environments 2 and 3 (shaded regions in Figure 3A, B). The inferior with sufficient moving capacity could persist by rapidly concentrating individuals in the higher-resource patch (Figure 4). When mobility of the superior consumer was high, the resource level in patch 1 always exceeded patch 2, and the potential advantage of the inferior reached zero (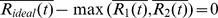; Figure 3A, B), preventing the inferior consumer from invading.

### Growth-dependent Dispersal

Growth-dependent dispersal enabled two consumers to coexist at steady states. Mathematical analyses suggested if the superior consumer exhibited very high mobility and the superior consumer and resource dynamics reached stable equilibrium, a sedentary inferior competitor had invasive potential (see Text S2). Mathematical analyses also indicated that the coexistence is possible because the superior consumer with very high mobility can also invade the stable equilibrium with the sedentary inferior competitor only. More specifically, we found that the ratio of minimal resource levels (R^*^_I, min_/R^*^_S, min_) and the ratio of carrying capacities (*k*_1_/*k*_2_) are the determinant of the coexistence of then. Numerical simulation confirmed this mathematical result in environment 1 (low carrying capacities; Figure 1G) and environment 2 (intermediate carrying capacities; Figure 1H). Coexistence occurred when the superior consumer moving capacity was high and the inferior consumer moving capacity was low, which was consistent with the coexistence realized by random dispersal. When superior moving capacity was high (Figure 1G, H), dispersal was maladaptive (Figure 2G,H). Coexistence occurred because maladaptive dispersal of the superior species was greater than that of the inferior species.

Growth-dependent dispersal also facilitated coexistence of the two consumers with fluctuating populations in environment 3 (high carrying capacities; region Cô in Figure 1I). Coexistence occurred under two conditions: moving capacity was low for the superior and high for the inferior consumer (top-left corner in Figure 1I, similar to fitness-dependent dispersal); and moving capacity was high for the superior and low for the inferior consumer (bottom-right corner in Figure 1I, similar to random dispersal). As can be seen in Figure 2I, the adaptiveness of growth-dependent dispersal of the inferior invader changed with the moving capacity of the superior patch resident. When the superior consumer moving capacity was low, dispersal was adaptive for the inferior (Figure 2I). Here, the potential fitness-dependent dispersal advantage (Figure 3C) was positive, indicating that resource levels were sometimes higher in patch 1 and sometimes higher in patch 2. Therefore, resource-tracking behavioris possible. Detailed community dynamics of this scenario is provided in the supplementary material (Text S6 and Figure S6, S7). Compared to the almost sedentary superior consumer, the inferior takes advantage of aggregated distribution in the currently higher patch (Figure S6). This distribution was not mainly caused by the net movement toward the currently better patch (Figure S6). Instead, net movement is toward a patch not long before it became the patch of higher resource level. In this way, growth-dependent dispersal performed the resource-tracking behavior similar to fitness-dependent dispersal.

However, under increased superior movement capacity, dispersal was maladaptive for the inferior (Figure 2I). Here, the potential fitness-dependent dispersal advantage was lost (Figure 3B); i.e., the resource level was always higher in one patch. The fast-moving superior had their net movement toward patch 2, resulting in lower ratio of its population stayed in patch 1 compared to the almost sedentary inferior. Thus, the predation pressure in patch 1 was relaxed, and the almost sedentary inferior could invade and coexist with the superior (bottom-right corner in Figure 1I). Detailed community dynamics of this scenario is provided in the supplementary material (Text S6 and Figure S6, S7).

## Discussion

The present study served to theoretically investigate the effects of directed dispersal on coexistence of competing consumers. Specifically, we identified the roles of dispersal in the trade-offs on which the coexistence mechanisms are based. Depending on adaptiveness, dispersal could have a disadvantage that should be compensated by competitive advantage, or an advantage that should offset competitive disadvantage, to promote coexistence. We detected two coexistence mechanisms correspond to the two cases, respectively. One mechanism was based on maladaptive movement of a superior consumer, consistent with the emigration-mediated coexistence concept. The second, a new mechanism, was derived from adaptive movement of an inferior consumer. During fluctuations in resource levels, adaptive movement facilitated tracking the higher resource level patch by the inferior, resulting in coexistence with the superior consumer. To our knowledge, this would be a new mechanism of dispersal-mediated coexistence promoted by adaptive movement. Summary of the two coexistence mechanisms is provided in Table 1.

**Table 1:** Summarizing the conditions and mechanisms of the two coexistence mechanisms.

Coexistence promoted by two different mechanisms		
**Adaptiveness of dispersal**	Maladaptive	Adaptive
**Mechanism (Trade-offs involved)**	The superior competitor also performs stronger maladaptive dispersal. (Emigration-mediated coexistence)	The inferior competitor has higher ability to perform adaptive dispersal (that allows population to track patches of higher resource level).
**Dispersal modes involved**	Random dispersal; Growth-dependent dispersal	Fitness-dependent dispersal; Growth-dependent dispersal
**Constraint on moving capacities**	*d*_max,*S*_>*d*_max,*I*_	*d*_max,*S*_<*d*_max,*I*_
**Whether coexistence at steady state is possible**	Yes	No

### Adaptiveness and Coexistence Mechanisms of Directed Dispersal

Directed dispersal can be adaptive by rapid migration to the higher-resource patch (resource-tracking behavior). This resource-tracking behavior results from fluctuating resource levels, which occurs in environments with intermediate and high carrying capacities. In these environments, fitness-dependent dispersal promotes coexistence when the inferior competitor moving capacity is high (region Co^∧^ in Figure 1E, F). In addition, the moving capacity for the superior competitor should be low; otherwise, resource levels will always increase in one patch (indicated by potential advantage, Figure 3A, B), and resource-tracking behavior will not occur. In previous models of one species population performing fitness-dependent dispersal, population asynchrony is viewed as a stabilizing factor that reduces total population fluctuation (e.g. [31], [32]). However, our results suggest that asynchrony in consumer population sizes is coupled with asynchrony in resource levels (as in [31], [32]), which could make the system more susceptible to the invasion of adaptive disperser. Our model demonstrates that with fitness-dependent dispersal, when the superior moving capacity is low, the dynamics of the resource is favorable to the invasion of competitor with high moving capacity. This would be a newly described mechanism of dispersal-mediated coexistence, based on the trade-off between competitive ability (of the superior) and the ability to perform adaptive dispersal (of the inferior).

This new mechanism can be distinguished from competition-colonization (CC) trade-offs [4], [7] by substantial differences in model assumptions, and in the mechanism through which dispersal can be adaptive. First, CC trade-off models consider colonization-extinction dynamics. Most models of CC trade-off assume that dispersal is very slow relative to local dynamics so that the state of patches is either occupied or empty (the patch-dynamic models, [4], [5], [6], [7]); other models of CC trade-off consider growth and (seed) dispersal at distinct stages, and the seed dispersal follows Poisson function that patches receive no seed are considered extinct ([8], [9],[10]). These features make these models somewhat like the patch-dynamic models because they also perform extinction and re-colonization dynamics. With this colonization-extinction dynamics, the better colonizer takes advantages through colonizing empty patches. Second, CC trade-off coexistence generally requires certain degree of competitive hierarchy ([42], [43], [44]). Finally, although colonization ability is an advantage in CC trade-off, “colonization” is adaptive by involving dispersal and fecundity [2], [43]. In our model, however, directed dispersal was adaptive only because consumers tracked a higher-resource patch. Random dispersal appeared adaptive only in environment 3 (high carrying capacities), when moving capacity of both competitors was low (bottom-left corner in Figure 1C), and is restrictive to a small parameter region. The mechanisms contributing to adaptive random dispersal typically involve unstable dynamics of environmental suitability [45] or of population [46] in the source patch. Here, we show that, directed dispersal with non-equilibrium resource dynamics can be adaptive much more easily than random dispersal.

Our results indicated that directed dispersal was not always adaptive. Growth-dependent dispersal, which was derived from local information, was sometimes maladaptive because net movement of emigration from patch 1 to patch 2 and from patch 2 to patch 1 (eqn. 7) are determined by both resource levels (*R*_1_ and *R*_2_) and population sizes (*C_N_*_,1_ and *C_N_*_,2_). Although per capita emigration rate was lower in the high-fitness patch, emigrant number was higher because the patch supported a larger population, resulting in net emigration from the high-fitness to lower-fitness patch. This superior competitor maladaptive net emigration released the inferior consumer from competitive pressures, and the population was able to persist. This process is called “emigration-mediated coexistence” [47],and was facilitated by random [12], [13], [15], [19] and directed dispersal that based on competitor and predator densities [19]. These densities are not reliable cues for emigration, because density varies temporally with dispersal [19].Our growth-dependent dispersal model demonstrated maladaptive directed dispersal potentially promoted emigration-mediated coexistence.

Our results showed that emigration-mediated coexistence was inconsistent with fitness-dependent dispersal. Instead, fitness-dependent dispersers reached a steady state denoted “ideal-free distribution (IFD)” (region S* in Figure 1D, E), where fitness is identical among patches, and individuals could not increase fitness by changing patches [21]. The idea that fitness dependent dispersal results in IFD is consistent with previous studies using different model frameworks [48], [49].In our model, when resource levels were equal in two patches, fitness-dependent dispersers remained sedentary (see eq. 5), resulting in adaptive neutrality and did not promote steady state coexistence (Figure 1C). This pattern was congruent with previous studies showing fitness-dependent dispersal exhibited no apparent effects on species coexistence [19], [36].

### Directed Dispersal: Modeled and in Natural Metacommunities

Fundamental metacommunity properties and general inferences were evaluated by building the model on simple assumptions. We assumed that the resource was sedentary, which was reasonable for a terrestrial ecosystem with a plant resource, or an aquatic ecosystem with clear boundaries (e.g., lakes, rock pools). In an aquatic ecosystem without well-defined boundaries, the resource is likely to disperse passively; and if the passive movement strength is relatively small, this model is suited for such systems.

We assumed no direct cost of dispersal, although directed dispersal is an active process, costing time and energy. These costs could undermine the advantages of directed dispersal. Small costs of dispersal relative to benefits are negligible during dispersal decisions. Alternatively, large costs may be incorporated into dispersal decisions. Individuals leave a patch only when the expected emigration benefit exceeds some threshold, consequently reducing movements that are not cost-effective.

The ability of organisms to know the conditions in other patches is essential for an “ideal” dispersal mode in our fitness-dependent dispersal model. In addition to direct detection of cues, another mechanism to obtain information of other patches is the performance of conspecific dispersers (i.e. public information [50], [51]). For example, emigration tendency may be affected by the condition of the immigrant [52]. However, if distant information is costly or unavailable, alternative directed dispersal modes might have evolved. Even without information about patch quality, dispersal could be more or less asymmetric because of different susceptibility to asymmetric dispersal vectors (e.g., wind, current). Considering competition of similar species, asymmetric dispersal could be adaptive and coexistence is possible with certain degree of asymmetric dispersal for each species [53]. Also, directed dispersal mode could be based on local information. Empirically, emigration rates might be affected by local information, such as resource availability (e.g. [54]), conspecific density [55], and the presence of predator (e.g. [56], [57]).Directed dispersal has been detected in many taxa [22], and our study suggested directed dispersal promoted coexistence when competing species differed in their ability to perform adaptive dispersal. Among potentially competing species, differences in dispersal rates have been observed, for example, in stoneflies [58], moths [59], and minnows [60]. In addition, dispersal in two coexisting salt marsh snails responded differently to food abundance [61], and two competing voles exhibited different dispersal patterns in an enclosure experiment [62]. These differences in dispersal rate and mode have potential to affect competition and diversity in metacommunities.

We proposed a framework to distinguish two coexistence mechanisms in natural metacommunities. Emigration-mediated coexistence may occur if the superior competitor has increased mobility. Alternatively, if the inferior competitor shows increased mobility, the role adaptive movement serves in promoting coexistence should be considered. Our results also strongly emphasize the importance of adaptive dispersal in metacommunity study and management, particularly for the spatial mechanisms of coexistence.

### Directed Dispersal as a Product of Evolution

We found that directed dispersal can be adaptive or maladaptive, depending on dispersal mode and environmental stability. However, directed dispersal is assumed active, and therefore should be a product of natural selection. We expected maladaptive directed dispersal strategies in natural systems would be outcompeted over evolutionary time. Our results also suggested that for the inferior competitor, potential fitness-dependent dispersal advantage depends on dispersal mode and moving capacity of the superior competitor (Figure 3). This result suggested that the optimal strategy or evolutionary steady state of one species would depend on the trait of its competitor. Other theoretical studies suggest that the evolutionary outcome of dispersal rate will be affected by the trait of predator [63], [64], [65]. The evolutionary “game” of directed dispersal in the context of competition is clearly of interest for further studies.

Our numerical simulations investigated a broad range of trait value combinations for two species to obtain as many competitive outcomes as possible. However, trait evolution in natural ecosystems may be restricted by trade-offs between competitive and dispersal capacity. For example, due to trade-offs in life history traits [66], [67], the maintenance of the body parts that resulting in increased mobility may reduce competitive ability. In addition, adjustments in dispersal capacity may have evolved at a cost in reduced competitive ability [68].These trade-offs might result in decreased mobility of a superior relative to inferior competitor, promoting coexistence when dispersal is adaptive, supporting our results. Further consideration of trade-offs will contribute to the extension of the original concept of coexistence mediated by CC trade-offs (e.g. [4], [7]), where trade-off intensity is critical for coexistence [44]. As we demonstrated, the integration of fitness-related cues can result in adaptive dispersal; and trade-offs between adaptive dispersal and competition can promote coexistence of competing species.

## Supporting Information

Figure S1 Average fitness differential of switching patches.
(EPS)

Figure S2 Community dynamics observed when the superior moving capacity was very high (*d*_max,*S*_ = 100), and the inferior was absent, with random dispersal and in environment 3.
(EPS)

Figure S3 Community dynamics observed when the superior moving capacity was very high (*d*_max,*S*_ = 100), with random dispersal and in environment 3.
(EPS)

Figure S4 Community dynamicsobservedwhen both consumers had low moving capacity, in environment 3 with random dispersal (bottom- left corner coexistence outcomes in Fig. 1C).
(EPS)

Figure S5 Alternative steady states observed at the transition between S^∧^ to I^∧^ in Figure 1C.
(EPS)

Figure S6 Community dynamics observed when the superior had low, and the inferior had high moving capacity, in environment 3 with growth-dependent dispersal (top- left corner coexistence outcomes in Fig 1 I).
(EPS)

Figure S7 Community dynamics observed when the superior had high, and the inferior had low moving capacity, in environment 3 with growth-dependent dispersal (bottom- right corner coexistence outcomes in Fig 1 I).
(EPS)

Text S1 Coexistence under fitness-dependent dispersal.
(DOCX)

Text S2 Coexistence under growth-dependent dispersal.
(DOCX)

Text S3 Processes of numerical simulation.
(DOCX)

Text S4 Fitness differential of switching patches.
(DOCX)

Text S5 Detailed community dynamics in the scenario with random dispersal in environment 3.
(DOCX)

Text S6 Detailed community dynamics in the scenario with growth-dependent dispersal in environment 3.
(DOCX)

## References

[1] Leibold MA, Holyoak M, Mouquet N, Amarasekare P, Chase JM, et al (2004) The metacommunity concept: a framework for multi-scale community ecology. Ecology Letters 7: 601–613.

[2] Amarasekare P (2003) Competitive coexistence in spatially structured environments: a synthesis. Ecology Letters 6: 1109–1122.

[3] Mouquet N, Hoopes MF, Amarasekare P (2005) The world is patchy and heterogeneous! Trade-off and source-sink dynamics in competitive metacommunities. In: Holyoak M, Leibold MA, Holt RD, editors. Metacommunities: Spatial Dynamics and Ecological Communities. Chicago, IL: University of Chicago Press. 237–262.

[4] Levins R, Culver D (1971) Regional coexistence of species and competition between rare species Proceedings of the National Academy of Sciences of the United States of America 68: 1246–&. 10.1073/pnas.68.6.1246 PMC389163 16591932

[5] Levin SA (1974) Dispersion and population interactions. American Naturalist 108: 207–228.

[6] Hanski I (1983) Coexistence of competitors in patchy environment. Ecology 64: 493–500.

[7] Tilman D (1994) Competition and biodiversity in spatially structured habitats. Ecology 75: 2–16.

[8] Skellam JG (1951) Random dispersal in theoretical populations. Biometrika 38: 196–218. 14848123

[9] Levine JM, Rees M (2002) Coexistence and relative abundance in annual plant assemblages: the roles of competition and colonization. The American naturalist 160: 452–467. 10.1086/342073 18707522

[10] Jansen VAA, Mulder GSEE (1999) Evolving biodiversity. Ecology Letters 2: 379–386.

[11] Pulliam HR (1988) Source, sinks, and population regulation American Naturalist. 132: 652–661.

[12] Takeuchi Y (1989) Diffusion-mediated persistence in two-species competition Lotka-Volterra model. Mathematical Biosciences 95: 65–83. 2520178 10.1016/0025-5564(89)90052-7

[13] Kishimoto K (1990) Coexistence of any number of species in the Lotka-Volterra competitive system over two patches. Theoretical Population Biology 38: 149–158.

[14] Amarasekare P, Nisbet RM (2001) Spatial heterogeneity, source-sink dynamics, and the local coexistence of competing species. American Naturalist 158: 572–584. 10.1086/323586 18707352

[15] Namba T, Hashimoto C (2004) Dispersal-mediated coexistence of competing predators. Theoretical Population Biology 66: 53–70. 15225575 10.1016/j.tpb.2004.03.003

[16] Cadotte M, Fortner A, Fukami T (2006) The effects of resource enrichment, dispersal, and predation on local and metacommunity structure. Oecologia 149: 150–157. 16639564 10.1007/s00442-006-0426-z

[17] Östman Ö, Kneitel JM, Chase JM (2006) Disturbance alters habitat isolation’s effect on biodiversity in aquatic microcosms. Oikos 114: 360–366.

[18] Cadotte MW (2006) Dispersal and species diversity: A meta-analysis. American Naturalist 167: 913–924. 10.1086/504850 16649154

[19] Amarasekare P (2010) Effect of non-random dispersal strategies on spatial coexistence mechanisms. Journal of Animal Ecology 79: 282–293. 19682160 10.1111/j.1365-2656.2009.01607.x

[20] Abrams PA, Wilson WG (2004) Coexistence of competitors in metacommunities due to spatial variation in resource growth rates; does R * predict the outcome of competition? Ecology Letters 7: 929–940.

[21] Fretwell SD, Lucas HL (1969) On territorial behavior and other factors influencing habitat distribution in birds. Acta Biotheoretica 19: 16–36.

[22] Bowler DE, Benton TG (2005) Causes and consequences of animal dispersal strategies: relating individual behaviour to spatial dynamics. Biological Reviews 80: 205–225. 15921049 10.1017/s1464793104006645

[23] Campbell CAM, Pettersson J, Pickett JA, Wadhams LJ, Woodcock CM (1993) Spring migration of damson-hop aphid, *Phorodon humuli* (Homoptera, Aphididae),and summer host plant-derived semiochemicals released on feeding. Journal of Chemical Ecology 19: 1569–1576. 24249183 10.1007/BF00984897

[24] Kostal V, Finch S (1996) Preference of the cabbage root fly, *Delia radicum* (L), for coloured traps: Influence of sex and physiological status of the flies, trap background and experimental design. Physiological Entomology 21: 123–130.

[25] France KE, Duffy JE (2006) Diversity and dispersal interactively affect predictability of ecosystem function. Nature 441: 1139–1143. 16810254 10.1038/nature04729

[26] Namba T (1980) Density-dependent dispersal and spatial distribution of a population. Journal of Theoretical Biology 86: 351–363. 7442297 10.1016/0022-5193(80)90011-9

[27] Gurney WSC, Nisbet RM (1975) The regulation of inhomogeneous populations. Journal of Theoretical Biology 52: 441–457. 1195750 10.1016/0022-5193(75)90011-9

[28] Namba T (1989) Competition for space in a heterogeneous environment. Journal of Mathematical Biology 27: 1–16.

[29] Shigesada N, Kawasaki K, Teramoto E (1979) Spatial segregation of interacting species. Journal of Theoretical Biology 79: 83–99. 513804 10.1016/0022-5193(79)90258-3

[30] Abrams PA (2000) The impact of habitat selection on the spatial heterogeneity of resource in varying environments. Ecology 81: 2902–2913.

[31] Abrams PA, Ruokolainen L (2011) How does adaptive consumer movement affect population dynamics in consumer–resource metacommunities with homogeneous patches? Journal of Theoretical Biology 277: 99–110. 21371481 10.1016/j.jtbi.2011.02.019

[32] Ruokolainen L, Abrams PA, McCann KS, Shuter BJ (2011) The roles of spatial heterogeneity and adaptive movement in stabilizing (or destabilizing) simple metacommunities. Journal of Theoretical Biology 291: 76–87. 21945147 10.1016/j.jtbi.2011.09.004

[33] Abrams PA, Ruokolainen L, Shuter BJ, McCann KS (2012) Harvesting creates ecological traps: consequences of invisible mortality risks in predator–prey metacommunities. Ecology 93: 281–293. 22624310 10.1890/11-0011.1

[34] Abrams PA (2007) Habitat choice in predator-prey systems: spatial instability due to interacting adaptive movements. The American naturalist 169: 581–594. 10.1086/512688 17427130

[35] Holt RD (1985) Popolation dynamics in 2-patch environments: some anomalous consequences of an optimal habitat distribution. Theoretical Population Biology 28: 181–208.

[36] Armsworth PR, Roughgarden JE (2005) The impact of directed versus random movement on population dynamics and biodiversity patterns. American Naturalist 165: 449–465. 10.1086/428595 15791537

[37] McPeek MA, Holt RD (1992) The evolution of dispersal in spatially and temporally varying environments. American Naturalist 140: 1010–1027.

[38] Tilman D (1982) Resource competition and community structure. Princeton, N.J.: Princeton University Press. 7162524

[39] Hsu SB, Hubbell SP, Waltman P (1978) Competing predators. SIAM Journal on Applied Mathematics 35: 617–625.

[40] Hsu SB, Hubbell SP, Waltman P (1978) A contribution to the theory of competing predators. Ecological Monographs 48: 337–349.

[41] Geritz SAH, Kisdi É, Meszéna G, Metz JAJ (1998) Evolutionarily singular strategies and the adaptive growth and branching of the evolutionary tree. Evolutionary Ecology: 35–57.

[42] Yu DW, Wilson HB (2001) The competition-colonization trade-off is dead; long live the competition-colonization trade-off. The American naturalist 158: 49–63. 10.1086/320865 18707314

[43] Bolker BM, Pacala SW (1999) Spatial moment equations for plant competition: Understanding spatial strategies and the advantages of short dispersal. American Naturalist 153: 575–602. 10.1086/303199 29585643

[44] Calcagno V, Mouquet N, Jarne P, David P (2006) Coexistence in a metacommunity: the competition–colonization trade-off is not dead. Ecology Letters 9: 897–907. 16913929 10.1111/j.1461-0248.2006.00930.x

[45] Jansen VAA, Yoshimura J (1998) Populations can persist in an environment consisting of sink habitats only. Proceedings of the National Academy of Sciences of the United States of America 95: 3696–3698. 9520428 10.1073/pnas.95.7.3696 PMC19898

[46] Holt RD (1997) On the evolutionary stability of sink populations. Evolutionary Ecology 11: 723–731.

[47] Amarasekare P (2008) Spatial dynamics of foodwebs. Annual Review of Ecology Evolution and Systematics 39: 479–500.

[48] Cosner C (2005) A dynamic model for the ideal-free distribution as a partial differential equation. Theoretical Population Biology 67: 101–108. 15713323 10.1016/j.tpb.2004.09.002

[49] Cantrell RS, Cosner C, Lou Y (2008) Approximating the ideal free distribution via reaction–diffusion–advection equations. Journal of Differential Equations 245: 3687–3703.

[50] Danchin E, Giraldeau LA, Valone TJ, Wagner RH (2004) Public information: From nosy neighbors to cultural evolution. Science 305: 487–491. 15273386 10.1126/science.1098254

[51] Clobert J, Le Galliard JF, Cote J, Meylan S, Massot M (2009) Informed dispersal, heterogeneity in animal dispersal syndromes and the dynamics of spatially structured populations. Ecology Letters 12: 197–209. 19170731 10.1111/j.1461-0248.2008.01267.x

[52] Cote J, Clobert J (2007) Social information and emigration: lessons from immigrants. Ecology Letters 10: 411–417. 17498140 10.1111/j.1461-0248.2007.01032.x

[53] Salomon Y, Connolly SR, Bode L (2010) Effects of asymmetric dispersal on the coexistence of competing species. Ecology Letters 13: 432–441. 20100238 10.1111/j.1461-0248.2009.01436.x

[54] Snider SB, Gilliam JF (2008) Movement ecology: Size-specific behavioral response of an invasive snail to food availability. Ecology 89: 1961–1971. 18705382 10.1890/07-0715.1

[55] Powers SP, Peterson CH (2000) Conditional density dependence: the flow trigger to expression of density-dependent emigration in Bay Scallops. Limnology and Oceanography 45: 727–732.

[56] Weisser WW, Braendle C, Minoretti N (1999) Predator-induced morphological shift in the pea aphid. Proceedings of the Royal Society of London Series B-Biological Sciences 266: 1175–1181.

[57] Poethke HJ, Weisser WW, Hovestadt T (2010) Predator-induced dispersal and the evolution of conditional dispersal in correlated environments. American Naturalist 175: 577–586. 10.1086/651595 20331365

[58] Richardson JS (1991) Seasonal food limitation of detritivores in a montane stream-an experimental test. Ecology 72: 873–887.

[59] Nieminen M (1996) Migration of moth species in a network of small islands. Oecologia 108: 643–651. 28307797 10.1007/BF00329038

[60] Schaefer J (2001) Riffles as barriers to interpool movement by three cyprinids (Notropis boops, Campostoma anomalum and Cyprinella venusta). Freshwater Biology 46: 379–388.

[61] Byers JE (2000) Effects of body size and resource availability on dispersal in a native and a non-native estuarine snail. Journal of Experimental Marine Biology and Ecology 248: 133–150. 10771298 10.1016/s0022-0981(00)00163-5

[62] Lin YTK, Batzli GO (2001) The influence of habitat quality on dispersal demography, and population dynamics of voles. Ecological Monographs 71: 245–275.

[63] Wang W, Takeuchi Y (2009) Adaptation of prey and predators between patches. Journal of Theoretical Biology 258: 603–613. 19254729 10.1016/j.jtbi.2009.02.014

[64] Pillai P, Gonzalez A, Loreau M (2012) Evolution of Dispersal in a Predator-Prey Metacommunity. The American naturalist 179: 204–216. 10.1086/663674 22218310

[65] Flaxman S, Lou Y, Meyer F (2011) Evolutionary ecology of movement by predators and prey. Theoretical Ecology 4: 255–267.

[66] Saglam IK, Roff DA, Fairbairn DJ (2008) Male sand crickets trade-off flight capability for reproductive potential. Journal of Evolutionary Biology 21: 997–1004. 18489565 10.1111/j.1420-9101.2008.01548.x

[67] Zhao LQ, Zhu DH, Zeng Y (2010) Physiological trade-offs between flight muscle and reproductive development in the wing-dimorphic cricket Velarifictorus ornatus. Entomologia Experimentalis Et Applicata 135: 288–294.

[68] Krug PJ, Zimmer RK (2000) Developmental dimorphism and expression of chemosensory-mediated behavior: Habitat selection by a specialist marine herbivore. Journal of Experimental Biology 203: 1741–1754. 10804164 10.1242/jeb.203.11.1741

