# Correction: Difference In Adaptive Dispersal Ability Can Promote Species Coexistence in Fluctuating Environments

**DOI:** 10.1371/annotation/c0358dfe-be57-4579-8b1d-6aa1f671eafc

**Published:** 2013-07-16

**Authors:** Wei-Ting Lin, Chih-hao Hsieh, Takeshi Miki

There is a typographical error in the title. The correct title is: Difference In Adaptive Dispersal Ability Can Promote Species Coexistence in Fluctuating Environments. The correct citation is: Lin W-T, Hsieh C-h, Miki T (2013) Difference In Adaptive Dispersal Ability Can Promote Species Coexistence in Fluctuating Environments. PLoS ONE 8(2): e55218. doi:10.1371/journal.pone.0055218 

