# Supplementary material for: Difference Inadaptive Dispersal Ability Can Promote Species Coexistence in Fluctuating Environments
Source: PLoS One. 2013 Feb 1;8(2):e55218. doi: 10.1371/journal.pone.0055218 (PMC3562337; doi:10.1371/journal.pone.0055218)
Supplement: Text S1 — Coexistence under fitness-dependent dispersal. (DOCX) [file pone.0055218.s008.docx]

Text S1 Coexistence under fitness-dependent dispersal

We analyzed the model of equation 2 with fitness-dependent dispersal (eq. 4, 5) for the coexistence criteria of two competing consumers. To make analysis tractable, we consider mutual invasibility of the two consumer species; that is, whether each species can invade the stable steady state where this species is absent. If two species are mutually invasible, it is likely that they can coexist. In this section, we first find out locally stable equilibrium of a sub-model with only one consumer. Then, we check the invasibility of the other consumer species to this sub-model. Finally we discuss the criteria for the mutual invasibility of the two species.

**Stable equilibria of sub-model**

Consider a sub-model with only resource species and one consumer species. Let *C_j_* denote the population of the resident consumer species in patch *j*. Let *e*, *h*, and *d_max_* represent the encounter rate, the handling time, and the moving ability of the resident consumer, respectively. By modifying equation 2, we have the following sub-model:

$$\begin{matrix} \frac{dR_{1}}{dt}=\left(1-\frac{R_{1}}{k_{1}}\right)R_{1}-\frac{eR_{1}}{1+ehR_{1}}C_{1}, \\ \frac{dR_{2}}{dt}=\left(1-\frac{R_{2}}{k_{2}}\right)R_{2}-\frac{eR_{2}}{1+ehR_{2}}C_{2}, \\ \frac{dC_{1}}{dt}=\left(\frac{eR_{1}}{1+ehR_{1}}-m\right)C_{1}+D, \\ \frac{dC_{2}}{dt}=\left(\frac{eR_{2}}{1+ehR_{2}}-m\right)C_{2}-D. \end{matrix}$$

Here, “*D*” is the net dispersal of the consumer from patch 2 to patch 1. The positive *D* indicates net movement from patch 2 to patch 1, whereas the negative *D* indicates net movement from patch 1 to patch 2. From eq. 5,

$$D=\left\{\begin{matrix} d_{\max}\left[\frac{(f_{1}-f_{2})}{1/h}\right]^{2}C_{2}\text{ if }f_{1}>f_{2}, \\ 0\text{ if }f_{1}=f_{2}, \\ -d_{\max}\left[\frac{(f_{1}-f_{2})}{1/h}\right]^{2}C_{1}\text{ if }f_{1}<f_{2}, \end{matrix}\right.$$

where *f_j_* is the per capita growth rate of *C_j_*, as follows,

$$f_{j}=\frac{eR_{j}}{1+ehR_{j}}-m.$$

There are seven equilibria: *E*_00_ (*C*_1_ = 0, *C*_2_ = 0, *R*_1_ = 0, *R*_2_ = 0), *E*_01_ (*C*_1_ = 0, *C*_2_ = 0, *R*_1_ = *k*_1_*, R*_2_ = *0*)*, E*_02_ (*C*_1_ = 0, *C*_2_ = 0, *R*_1_ = 0, *R*_2_ = *k*_2_)*, E*_0_ (*C*_1_ = 0, *C*_2_ = 0, *R*_1_ = *k*_1_*, R*_2_ = *k*_2_), *E*_1_ (*C*_1_ > 0, *C*_2_ = 0, *R*_1_ > 0, *R*_2_ = 0), *E*_2_ (*C*_1_ = 0, *C*_2_ > 0, *R*_1_ = 0, *R*_2_ > 0), and *E*_3_ (*C*_1_ *> 0, C*_2_ > 0, *R*_1_ > 0, *R*_2_ > 0).

Among these equilibria, *E*_00_, *E*_01_, and *E*_02_ are locally unstable because *R*_1_ and/or *R*_2_ that is absent can invade the equilibrium in the absence of consumers (*C*_1_***=*C*_2_*** = 0) as follows,

$$\begin{matrix} \lim_{R_{1}\to0} \frac{1}{R_{1}}\frac{dR_{1}}{dt}=\lim_{R_{1}\to0} \frac{1}{R_{1}}(1-\frac{R_{1}}{k_{1}})R_{1} \\ =1>0,\text{ }when\text{ }C_{1}=0,\text{ }and \\ \\ \lim_{R_{2}\to0} \frac{1}{R_{2}}\frac{dR_{2}}{dt}=\lim_{R_{2}\to0} \frac{1}{R_{2}}(1-\frac{R_{2}}{k_{2}})R_{2} \\ =1>0,\text{ }when\text{ }C_{2}=0. \end{matrix}$$

At *E*_0_, net dispersal of the consumer is zero (*D**= 0) because *C*_1_*** = 0, *C*_2_***= 0. *E_0_* is unstable if the consumer can invade in at least one patch. Without lost of generality, we assumed patch 1 has higher carrying capacity (*k*_1_ >*k*_2_). Then, *E*_0_ is unstable as long as the consumer can invade patch 1. This condition is equivalent to

$$\begin{matrix} \lim_{\text{C}_{1}\to0} \frac{1}{C_{1}}\frac{dC_{1}}{dt}=\frac{ek_{1}}{1+ehk_{1}}-m>0, \\ \Leftrightarrow k_{1}\text{>}\frac{m}{e(1-mh)}. \end{matrix}$$

This implies that the consumer can invade if the carrying capacity is high enough.

At *E*_1_ (*C*_1_ > 0, *C*_2_ = 0, *R*_1_ > 0, *R*_2_ = 0), since fitness of the consumer at patch 1 is higher, the dispersal function is

$$\begin{matrix} D\text{*}=d_{\max}\left[\frac{(f_{1}\text{*}-f_{2}\text{*})}{1/h}\right]^{2}C_{2}\text{*} \\ =0, \end{matrix}$$

*R*_2_ can invade and *E*_1_ is locally unstable because

$$\begin{matrix} \lim_{R_{2}\to0} \frac{1}{R_{2}}\frac{dR_{2}}{dt}=\lim_{R_{2}\to0} (1-\frac{R_{2}}{k_{2}}) \\ \text{=1>0.} \end{matrix}$$

As *E*_1_ is locally unstable, similarly *E*_2_ is locally unstable because of its symmetry to *E*_1_.

At *E*_3_, we have

Here, *f*_1_* and *f*_2_* denote the fitness (per capita growth rate) of the consumer in patch 1 and patch 2, respectively, at *E*_3_. At *E*_3_, per capita growth rates are

$$\text{ }f\text{*}_{1}=\frac{-D\text{*}}{C_{1}\text{*}},\text{ }f_{2}\text{*}=\frac{D\text{*}}{C_{2}\text{*}}.$$

We can assume *R*_1_*** ≥ *R*_2_*** without lost of generality at *E*_3_. Then *D**, net movement from patch 2 to patch 1, is non-negative because the fitness in patch 1 is no less than the fitness in patch 2. Therefore, the solution should satisfy

$$\left\{\begin{matrix} R_{1}\text{*}\geq R_{2}\text{*}\Leftrightarrow f_{1}\text{*}\geq f_{2}\text{*,} \\ \begin{matrix} f_{1}\text{*}=\frac{-D\text{*}}{C_{1}\text{*}}\leq0, \\ f_{2}\text{*}=\frac{D\text{*}}{C_{2}\text{*}}\geq0. \end{matrix} \end{matrix}\right.$$

Then the unique solution is *R*_1_***=*R*_2_*** and *D**= 0. This result means that the resource levels are equal in two patches and there is no net movement between two patches at the unique equilibrium with positive *C*_1_*** and *C*_2_*** under fitness-dependent dispersal. At *E*_3_, the densities of four populations are equivalent to those in the absence of dispersal, as follows,

$$\begin{matrix} R_{1}\text{*}=R_{2}\text{*}=\frac{m}{e(1-mh)}, \\ C_{1}\text{*}=\frac{1+ehR_{1}\text{*}}{e}(1-\frac{R_{1}\text{*}}{k_{1}}), \\ C_{2}\text{*}=\frac{1+ehR_{2}\text{*}}{e}(1-\frac{R_{2}\text{*}}{k_{2}}). \end{matrix}$$

Next, we checked the local stability of *E*_3_ by focusing on the following Jacobian matrix,

$$J_{3}=\left[\begin{matrix} 1-\frac{2}{k_{1}}R_{1}\text{*}-\frac{eC_{1}\text{*}}{(1+ehR_{1}\text{*})^{2}} & 0 & \frac{-eR_{1}\text{*}}{1+ehR_{1}\text{*}} & 0 \\ 0 & 1-\frac{2}{k_{2}}R_{2}\text{*}-\frac{eC_{2}\text{*}}{(1+ehR_{2}\text{*)}^{2}} & 0 & \frac{-eR_{2}\text{*}}{1+ehR_{2}\text{*}} \\ \frac{eC_{1}\text{*}}{(1+ehR_{1}\text{*)}^{2}}+\frac{\partial}{\partial R_{1}}D\text{*} & \frac{\partial}{\partial R_{2}}D\text{*} & \frac{eR_{1}\text{*}}{1+ehR_{1}\text{*}}-m+\frac{\partial}{\partial C_{1}}D\text{*} & \frac{\partial}{\partial C_{2}}D\text{*} \\ -\frac{\partial}{\partial R_{1}}D\text{*} & \frac{eC_{2}\text{*}}{(1+ehR_{2}\text{*)}^{2}}-\frac{\partial}{\partial R_{2}}D\text{*} & -\frac{\partial}{\partial C_{1}}D\text{*} & \frac{eR_{2}\text{*}}{1+ehR_{2}\text{*}}-m-\frac{\partial}{\partial C_{2}}D\text{*} \end{matrix}\right].$$

At *E*_3_, *f*_1_ *= f*_2_. Therefore we have

$$\left.\frac{\partial D}{\partial R_{1}}\right|_{E_{3}}=\left.\frac{\partial D}{\partial R_{2}}\right|_{E_{3}}=\left.\frac{\partial D}{\partial C_{1}}\right|_{E_{3}}=\left.\frac{\partial D}{\partial C_{2}}\right|_{E_{3}}=0.$$

We finally have the following Jacobian matrix evaluated at the equilibrium *E*_3_,

$$J_{3}=\left[\begin{matrix} -\frac{R_{1}\text{*}}{k_{1}}+mh(1-\frac{R_{1}\text{*}}{k_{1}}) & 0 & -m & 0 \\ 0 & -\frac{R_{2}\text{*}}{k_{2}}+mh(1-\frac{R_{2}\text{*}}{k_{2}}) & 0 & -m \\ \frac{eC_{1}\text{*}}{\left(1+ehR_{1}\text{*}\right)^{2}} & 0 & 0 & 0 \\ 0 & \frac{eC_{2}\text{*}}{\left(1+ehR_{2}\text{*}\right)^{2}} & 0 & 0 \end{matrix}\right].$$

The characteristic equation for *J*_3_ is,

$$(\lambda^{2}-A\lambda+mE)(\lambda^{2}-B\lambda+mF)=0,$$

where

$$\begin{matrix} A=-\frac{R_{1}*}{k_{1}}+mh(1-\frac{R_{1}*}{k_{1}}), \\ B=-\frac{R_{2}*}{k_{2}}+mh(1-\frac{R_{2}*}{k_{2}}), \\ E=\frac{eC_{1}*}{\left(1+ehR_{1}*\right)^{2}}, \\ F=\frac{eC_{2}*}{\left(1+ehR_{2}*\right)^{2}}, \end{matrix}$$

where, *E* and *F* are always positive. The conditions for real part of all eigenvalues to be negative are *–A* > 0*,* which is equivalent to

$$k_{1}<\frac{(mh+1)}{eh(1-mh)},\text{ }$$

and *–B* > 0*,* which is equivalent to

$$k_{2}<\frac{(mh+1)}{eh(1-mh)}.$$

Therefore, *E*_3_ is locally stable when:

$$k_{1}<\frac{(1+mh)}{eh(1-mh)},\text{ and }k_{2}<\frac{(1+mh)}{eh(1-mh)}.$$

Note that if there is no dispersal, the local stability of non-trivial steady state (*C** > 0, *R** > 0) in a patch with carrying capacity *k* requires that $k<\frac{(1+mh)}{eh(1-mh)}$. Thus, the criterion for *E*_3_ to be locally stable is that *C-R* equilibria are locally stable in both patches in the absence of dispersal.

We found that the unique stable equilibrium with positive consumer population is *E*_3_. Then, we will check the criteria for invasibility of another consumer when *E*_3_ is locally stable in this sub-model.

***Invasibility of another consumer***

Recall that at *E*_3_, *R*_1_ and *R*_2_ are kept at the critical resource level of the resident consumer; that is,

$$R_{1}\text{*}=R_{2}\text{*}=\frac{m}{e(1-mh)}.$$

A sedentary invader can invade *E*_3_ if and only if the invader has a lower minimal resource level (*R*_min_*), which means that the invading consumer is a superior competitor to the resident consumer.

When the resident consumer adopts fitness-dependent dispersal, the stable equilibrium is realized only when *R*_1_***=*R*_2_***. Consider an invader adopting fitness dependent dispersal. The dispersal function of the invader to this equilibrium is zero; that is, there is no movement between patches. Therefore, the condition for an invader adopting fitness-dependent dispersal is the same as in the case of the sedentary invader, which is, if and only if the invader is the superior (i.e. has a lower *R*_min_* value).

***Coexistence criteria***

We found that irrespective of moving ability, the invader can invade if and only if it is a superior competitor to the resident species. It follows that, the mutual invasibility is not possible and that coexistence at steady state cannot be realized under the fitness-dependent dispersal.
