# Supplementary material for: Difference Inadaptive Dispersal Ability Can Promote Species Coexistence in Fluctuating Environments
Source: PLoS One. 2013 Feb 1;8(2):e55218. doi: 10.1371/journal.pone.0055218 (PMC3562337; doi:10.1371/journal.pone.0055218)
Supplement: Text S2 — Coexistence under growth-dependent dispersal. (DOCX) [file pone.0055218.s009.docx]

Text S2 Coexistence under growth-dependent dispersal

We focused on the model of equation 2 with the growth-dependent dispersal (by incorporating eq. 7) and investigate the coexistence criteria of two competing consumers. To make analysis tractable, we consider mutual invasibility of the two consumer species; that is, whether each species can invade the stable steady state where this species is absent. If two species are mutually invasible, the coexistence can be realized. For analytical tractability, we considered a limiting case where dispersal is very large and then we can apply the aggregation method [[1](#_ENREF_1),[2](#_ENREF_2)] for simplifying the sub-model.

The simplified sub-model is applicable to the invader and/or the resident consumer species. When invader disperses very fast, we use the simplified sub-model to calculate its invasibility. When resident disperses very fast, we find out locally stable equilibrium of the simplified sub-model, and check invasibility of the other consumer species to this sub-model. Finally we derive the criteria of the mutual invasibility for three conditions: sedentary invader and fast-moving resident, fast-moving invader and sedentary resident, or fast-moving invader and fast-moving resident.

**Sub-model**

Consider a sub-model with only resource species and one consumer species. The population of the resident consumer species in patch *j* is denoted by *C_j_*. Let *e*, *h*, and *d_max_* denote the encounter rate, the handling time, and the moving ability of the resident consumer, respectively. By Modifying equation 2, we have the following sub-model:

$$\begin{matrix} \frac{dR_{1}}{dt}=(1-\frac{R_{1}}{k_{1}})R_{1}-\frac{eR_{1}}{1+ehR_{1}}C_{1} \\ \frac{dR_{2}}{dt}=(1-\frac{R_{2}}{k_{2}})R_{2}-\frac{eR_{2}}{1+ehR_{2}}C_{2} \\ \frac{dC_{1}}{dt}=(\frac{eR_{1}}{1+ehR_{1}}-m)C_{1}+d_{\max}\left[(\frac{1}{1+ehR_{2}})C_{2}-(\frac{1}{1+ehR_{1}})C_{1}\right] \\ \frac{dC_{2}}{dt}=(\frac{eR_{2}}{1+ehR_{2}}-m)C_{2}+d_{\max}\left[(\frac{1}{1+ehR_{1}})C_{1}-(\frac{1}{1+ehR_{2}})C_{2}\right] \end{matrix}$$

***Stable equilibria of reduced model: using aggregation method***

Then, we apply the aggregation method [[1](#_ENREF_1),[2](#_ENREF_2)] to reduce the model. We assumed the moving ability of the consumer is very high; that is, *d_max_* is very large. Thus, the dispersal part is much faster than the growth part, and it always reaches equilibrium in the slow time-scale where population dynamics take place. As the dispersal part reaches equilibrium, we have

$$(\frac{1}{1+ehR_{2}})C_{2}-(\frac{1}{1+ehR_{1}})C_{1}\text{=0.}$$

Now we introduce a new variable, *C* to describe the total population. *C*_1_ and *C*_2_ can be replaced by *C*,

$$\begin{matrix} C=C_{1}+C_{2}, \\ C_{1}=\frac{1+ehR_{1}}{2+eh(R_{1}+R_{2})}C, \\ C_{2}=\frac{1+ehR_{2}}{2+eh(R_{1}+R_{2})}C. \end{matrix}$$

Then, the original model is reduced to the three-dimensional model,

$$\begin{matrix} \frac{dC}{dt}=\left[\frac{e(R_{1}+R_{2})}{2+eh(R_{1}+R_{2})}\right]C-mC\equiv g_{1}, \\ \frac{dR_{1}}{dt}=R_{1}(1-\frac{R_{1}}{k_{1}})-\frac{eR_{1}}{2+eh(R_{1}+R_{2})}C\equiv g_{2}, \\ \frac{dR_{2}}{dt}=R_{2}(1-\frac{R_{2}}{k_{2}})-\frac{eR_{2}}{2+eh(R_{1}+R_{2})}C\equiv g_{3}. \end{matrix}$$

There are seven equilibria in the three-dimensional model: *E*_00_ (*C* = 0, *R*_1_ = 0, *R*_2_ = 0), *E*_01_ (*C* = 0, *R*_1_ = *k*_1_, *R*_2_ = 0), *E*_02_ (*C* = 0, *R*_1_ = 0, *R*_2_ = *k*_2_), *E*_0_ (*C* = 0, *R*_1_ = *k*_1_*, R*_2_ = *k*_2_), *E*_1_ (*C* > *0, R*_1_ > *0, R*_2_ = 0), *E*_2_ (*C* > 0, *R*_1_ = 0, *R*_2_ *> 0*), and *E*_3_ (*C* > 0, *R*_1_ > 0, *R*_2_ > 0).

In order to determine local stability of these equilibria, we need to check the Jacobian matrix at equilibrium points. The Jacobian matrix for the simplified three-dimensional model is

$$J=\left[\begin{matrix} \frac{e(R_{1}+R_{2})}{2+eh(R_{1}+R_{2})}-m & \frac{2eC}{\left[2+eh(R_{1}+R_{2})\right]^{2}} & \frac{2eC}{\left[2+eh(R_{1}+R_{2})\right]^{2}} \\ \frac{-eR_{1}}{2+eh(R_{1}+R_{2})} & 1-\frac{2R_{1}}{k_{1}}-eC\frac{2+ehR_{2}}{\left[2+eh(R_{1}+R_{2})\right]^{2}} & \frac{e^{2}hCR_{1}}{\left[2+eh(R_{1}+R_{2})\right]^{2}} \\ \frac{-eR_{2}}{2+eh(R_{1}+R_{2})} & \frac{e^{2}hCR_{2}}{\left[2+eh(R_{1}+R_{2})\right]^{2}} & 1-\frac{2R_{2}}{k_{2}}-eC\frac{2+ehR_{1}}{\left[2+eh(R_{1}+R_{2})\right]^{2}} \end{matrix}\right].$$

The Jacobian matrix evaluated at *E*_00_(*C* = 0, *R*_1_ = 0, *R*_2_ = 0) is

$$J_{00}=\left[\begin{matrix} -m & 0 & 0 \\ 0 & 1 & 0 \\ 0 & 0 & 1 \end{matrix}\right].$$

*E*_00_ is locally unstable because its eigenvalues are *–m*, 1, and 1.

The Jacobian matrix evaluated at *E*_01_(*C* = 0, *R*_1_ = *k*_1_, *R*_2_ = 0) is

$$J_{01}=\left[\begin{matrix} \frac{ek_{1}}{2+ehk_{1}}-m & 0 & 0 \\ \frac{-ek_{1}}{2+ehk_{1}} & -1 & 0 \\ 0 & 0 & 1 \end{matrix}\right].$$

*E*_01_ is locally unstable because its eigenvalues are $\frac{ek_{1}}{2+ehk_{1}}-m$, −1, and 1. *E*_02_ is also locally unstable because of its symmetry to *E*_01_.

At *E*_0_(*C* = 0, *R*_1_ = *k*_1_*, R*_2_ = *k*_2_), the Jacobian matrix is:

$$J_{0}=\left[\begin{matrix} \frac{e(k_{1}+k_{2})}{2+eh(k_{1}+k_{2})}-m & 0 & 0 \\ \frac{-ek_{1}}{2+eh(k_{1}+k_{2})} & -1 & 0 \\ \frac{-ek_{2}}{2+eh(k_{1}+k_{2})} & 0 & -1 \end{matrix}\right],$$

and the eigenvalues are $\frac{e(k_{1}+k_{2})}{2+eh(k_{1}+k_{2})}-m$, −1 and −1. *E*_0_ is locally stable when the real parts of all eigenvalues are negative, that is,

$$\frac{k_{1}+k_{2}}{2}<\frac{m}{e(1-mh)}.$$

Therefore, *E*_0_ is locally stable when the average of carrying capacities in patch 1 and patch 2 is smaller than the minimal resource level of the consumer.

At *E*_1_, the equilibrium values are

$$\begin{matrix} C\text{*}=\frac{R_{1}\text{*}}{m}(1-\frac{R_{1}*}{k_{1}}), \\ R_{1}\text{*}=\frac{2m}{e(1-mh)}, \\ R_{2}\text{*}=0. \end{matrix}$$

For checking its local stability, we derived the Jacobian matrix at *E*_1_ as follows,

$$J_{1}=\left[\begin{matrix} 0 & \frac{2eC*}{(2+ehR_{1}*)^{2}} & \frac{2eC*}{(2+ehR_{1}*)^{2}} \\ -m & 1-\frac{2R_{1}*}{k_{1}}-\frac{2eC*}{(2+ehR_{1}*)^{2}} & \frac{e^{2}hC*R_{1}*}{\text{(}2+ehR_{1}*\text{)}^{2}} \\ 0 & 0 & 1-\frac{eC*}{2+ehR_{1}*} \end{matrix}\right].$$

The characteristic equation for *J*_1_ is

$$\lambda^{3}-(E+I)\lambda^{2}+(EI-BD)\lambda+BDI=0,$$

where

$$\begin{matrix} B=\frac{2eC*}{(2+ehR_{1}*)^{2}} \\ D=-m \\ E=1-\frac{2R_{1}*}{k_{1}}-\frac{2eC*}{(2+ehR_{1}*)^{2}} \\ I=1-\frac{eC*}{2+ehR_{1}*}. \end{matrix}$$

The Routh-Hurwitz conditions [[3](#_ENREF_3)] for real part of all the eigenvalues to be negative are

$$\left\{\begin{matrix} -(E+I)>0, \\ EI-BD>0, \\ BDI>0, \\ -(E+I)\times(EI-BD)-BDI>0, \end{matrix}\right.$$

noting that

$$-(E+I)\text{=}-mh(1-\frac{R_{1}\text{*}}{k_{1}})<0.$$

It follows that the real parts of eigenvalues are not all negative. This means that *E*_1_ is always locally unstable. Similarly, *E*_2_ is locally unstable because of its symmetry to *E*_1_. This sub-model has no stable equilibrium where the resource level is zero in one of the two patches.

At *E*_3_, *C**> 0, *R*_1_*** > 0, and *R*_2_***> 0. Therefore,

$$\begin{matrix} C\text{*}=\frac{2+eh(R_{1}\text{*}+R_{2}\text{*})}{e}(1-\frac{R_{1}\text{*}}{k_{1}}), \\ R_{1}\text{*}=\frac{2m}{e(1-mh)}\frac{k_{1}}{k_{1}+k_{2}}, \\ R_{2}\text{*}=\frac{2m}{e(1-mh)}\frac{k_{2}}{k_{1}+k_{2}}. \end{matrix}$$

The Jacobian matrix evaluated at *E*_3_ is

$$J_{3}=\left[\begin{matrix} 0 & \frac{2eC\text{*}}{\left[2+eh(R_{1}\text{*}+R_{2}\text{*})\right]^{2}} & \frac{2eC\text{*}}{\left[2+eh(R_{1}\text{*}+R_{2}\text{*})\right]^{2}} \\ \frac{-eR_{1}\text{*}}{2+eh(R_{1}\text{*}+R_{2}\text{*})} & 1-\frac{2R_{1}\text{*}}{k_{1}}-eC\text{*}\frac{2+ehR_{2}\text{*}}{\left[2+eh(R_{1}\text{*}+R_{2}\text{*})\right]^{2}} & \frac{e^{2}hR_{1}*C*}{\left[2+eh(R_{1}*+R_{2}*)\right]^{2}} \\ \frac{-eR_{2}\text{*}}{2+eh(R_{1}\text{*}+R_{2}\text{*})} & \frac{e^{2}hR_{2}\text{*}C\text{*}}{\left[2+eh(R_{1}\text{*}+R_{2}\text{*})\right]^{2}} & 1-\frac{2R_{2}\text{*}}{k_{2}}-eC\text{*}\frac{2+ehR_{1}\text{*}}{\left[2+eh(R_{1}\text{*}+R_{2}\text{*})\right]^{2}} \end{matrix}\right].$$

The characteristic equation for this Jacobian matrix is:

$$(\lambda+\hat{A})(\lambda^{2}+\hat{B}\lambda+\hat{C})\text{=0,}$$

where

$$\begin{matrix} \hat{A}=\frac{R_{1}\text{*}}{k_{1}}, \\ \hat{B}=\frac{2m(1+mh)}{e(1-mh)}\frac{1}{k_{1}+k_{2}}-mh, \\ \hat{C}=\frac{2eC\text{*}}{\left[2+eh(R_{1}\text{*}+R_{2}\text{*})\right]^{2}}m. \end{matrix}$$

For real part of all eigenvalues to be negative it requires that $\hat{A}>0,\text{ }\hat{B}>0$, and$\hat{\text{C}}>0$, which are equivalent to

$$\frac{k_{1}+k_{2}}{2}<\frac{(1+mh)}{eh(1-mh)}.$$

When this condition is satisfied, *E*_3_ is locally stable. Note that the r.h.s. of the above inequality is the upper limit of carrying capacity (*k*) for stable *C-R* coexistence equilibrium in a one-patch model.

We found that the only stable equilibrium with positive consumer population is *E*_3_. Then we will check the criteria for invasibility of another consumer species to *E*_3_.

***Invasibility analysis for two consumer species***

Assuming that consumer *C* has high moving ability and that *N* is a competing consumer of *C*, we analyzed invasibility for three cases, (1) sedentary invader (*N*) invades *C-R* metacommunity at equilibrium, (2) fast-moving invader (*C*) invades *N-R* metacommunity at equilibrium, where *N* is a sedentary consumer, and (3) fast-moving invader (*N*) invades *C-R* metacommunity at equilibrium.

1. At equilibrium of fast moving *C* where *C* > 0, we consider the invasibility of the sedentary competitor *N* (for a competitor that can be superior or inferior). The encounter rate and handling time of this invading competitor *N* are denoted as *e_N_* and *h_N_*, respectively. If *N* can invade at least one patch, we define it as a successful invasibility of *N*.

The unique stable equilibrium of *C-R* dynamics is *E*_3_, where

$$R_{1}^{*}=\frac{2m}{e(1-mh)}\frac{k_{1}}{k_{1}+k_{2}},\text{ }R_{2}^{*}=\frac{2m}{e(1-mh)}\frac{k_{2}}{k_{1}+k_{2}}.$$

Without lost of generality, we assume *k*_1_ > *k*_2_; it follows that *R*_1_*** > *R*_2_***. Thus if *N* can invade patch 1, we consider it can invade this *C-R* metacommunity. If *N* cannot invade patch 1, it certainly cannot invade patch 2, where resource level is even lower.

We can calculate per capita growth rate of *N* when population is very small, as follows,

$$\lim_{N\to0} \frac{1}{N}\frac{dN}{dt}=\frac{e_{N}R_{1}^{*}}{1+e_{N}h_{N}R_{1}^{*}}-m.$$

For *N* to invade, this value should be positive. We can find the condition of *R*_1_*** with which *N* can invade.

$$\begin{matrix} \lim_{N\to0} \frac{1}{N}\frac{dN}{dt}=\frac{e_{N}R_{1}^{*}}{1+e_{N}h_{N}R_{1}^{*}}-m>0, \\ R_{1}^{*}>\frac{m}{e_{N}(1-mh_{N})}. \end{matrix}$$

Using *R*_1_*** value at *E*_3_, we have the following condition,

$$\frac{2k_{1}}{k_{1}+k_{2}}=\frac{2\frac{k_{1}}{k_{2}}}{1+\frac{k_{1}}{k_{2}}}>\frac{\frac{m}{e_{N}(1-mh_{N})}}{\frac{m}{e(1-mh)}}.$$

Note that $\frac{m}{e(1-mh)}$ is the minimal resource level of consumer *C* (*R*_min,C_*), and that$\frac{m}{e_{\text{N}}(1-mh_{\text{N}})}$ is the minimal resource level of the invading consumer *N* (*R*_min,N_*). We define the superior consumer as the one that has smaller *R*_min_* value. From this result, we conclude that a superior invader can always invade. However, it is still possible that an inferior competitor can invade. This happens when the ratio of *R*_min_* values of the inferior to that of the superior is small enough, and when *k*_1_/*k*_2_ is rather large. Large *k*_1_/*k*_2_ means the patches are rather heterogeneous because we assume *k*_1_>*k*_2_ (without lost of generality).

1. We consider the second case where consumer *C* that has high moving ability tries to invade the metacommunity of only a sedentary consumer *N* and resource *R* at equilibrium.

At this *N-R* equilibrium,

$$R_{1}^{*}=R_{2}^{*}=\frac{m}{e_{\text{N}}(1-mh_{\text{N}})}.$$

For successful invasibility of the invader *C*, the per capita growth rate of *C* should be positive. This condition is

$$\begin{matrix} \lim_{N\to0} \frac{1}{C}\frac{dC}{dt}\text{=}(\frac{e(R_{1}\text{*}+R_{2}\text{*})}{2+eh(R_{1}\text{*}+R_{2}\text{*})})-m>0 \\ \Leftrightarrow\frac{m}{e_{N}(1-mh_{N})}>\frac{m}{e(1-mh)} \\ \Leftrightarrow R_{\min,N}^{*}>R_{\min,C}^{*} \end{matrix}$$

Therefore, *C* can invade *N-R* metacommunity at equilibrium if and only if *C* is a superior competitor compare to *N*.

1. For a third case, both the invading consumer *N* and the resident consumer *C* have high moving ability. We checked the invasibility of *N* to *C-R* metacommunity.

At *C-R* stable equilibrium,

$$R_{1}^{*}=\frac{2m}{e(1-mh)}\frac{k_{1}}{k_{1}+k_{2}},\text{ }R_{2}^{*}=\frac{2m}{e(1-mh)}\frac{k_{2}}{k_{1}+k_{2}}.$$

For successful invasibility of the invader *N*, the per capita growth rate of *N* should be positive. This condition is

$$\begin{matrix} \lim_{N\to0} \frac{1}{N}\frac{dN}{dt}\text{=}\frac{e_{N}\text{(}R_{1}^{*}\text{+}R_{2}^{*}\text{)}}{2+e_{N}h_{N}\text{(}R_{1}^{*}\text{+}R_{2}^{*}\text{)}}-m>0 \\ \Leftrightarrow\frac{m}{e(1-mh)}>\frac{m}{e_{\text{N}}(1-mh_{\text{N}})}. \end{matrix}$$

Therefore, *N* can invade *C-R* stable equilibrium if and only if *N* is superior to *C*.

***Coexistence criteria***

Summing up invasibility criteria from the above three conditions, a superior competitor can always invade the steady states with an inferior competitor only. On the other hand, an inferior competitor can invade the steady states with a superior competitor only when the inferior invader is sedentary and the superior resident has very high moving ability. Then, mutual invasibility is realized and coexistence of two competing consumers is possible only when the inferior is sedentary and the superior moves very fast, and when patches are rather heterogeneous (i.e. *k*_1_/*k*_2_ is rather large).

**References**

1. Auger P, Poggiale JC (1996) Emergence of population growth models: Fast migration and slow growth. Journal of Theoretical Biology 182: 99-108.

2. Michalski J, Poggiale JC, Arditi R, Auger PM (1997) Macroscopic dynamic effects of migrations in patchy predator-prey systems. Journal of Theoretical Biology 185: 459-474.

3. Murray JD (2002) Mathematical biology. New York: Springer.
