# Supplementary material for: Difference Inadaptive Dispersal Ability Can Promote Species Coexistence in Fluctuating Environments
Source: PLoS One. 2013 Feb 1;8(2):e55218. doi: 10.1371/journal.pone.0055218 (PMC3562337; doi:10.1371/journal.pone.0055218)
Supplement: Text S3 — Processes of numerical simulation. (DOCX) [file pone.0055218.s010.docx]

**Text S3 Processes of numerical simulation**

**Simulation for competitive outcomes**

We carried out simulations using fourth-order Runge-Kutta method with a 0.001 step length in C language [[1](#_ENREF_1)], following these steps:

First, let a sub-model with only the superior consumer and the resource (hereafter, *C_S_-R* system) reaches its long-term dynamics by simulating 1,500 units of time. Initial value of resource populations are at carrying capacities, and the superior consumer populations are at 0.001 in each patch.

Second, introduce small populations of the inferior as invader (0.001 for each patch, totally 0.002) to the *C_S_-R* system. If the *C_S_-R* system before invasion has non-equilibrium dynamics, invader population is uniformly distributed across an invasion stage of 200 time steps.

Third, after invasion, we run for 30,000 units of time to obtain the long-term consequences of competition. We check the average populations and c.v. (coefficient of variation) of all at *t* = 9,000~10,000 and *t* = 19,000~20,000, and stop simulation if the inferior invader goes extinct (average population of the inferior < 0.001) or if equilibrium had been reached (average c.v. of all survived populations < 0.01). This simulation time is long enough for the model of most scenarios to reach reliable long-term outcomes (i.e., the qualitative result in Figure 1 will not change with even longer simulation time). In the scenario of random dispersal and environment 3 (corresponding to Figure 1G), however, the simulation time should be much longer because it is possible for the inferior competitor to exclude the superior competitor eventually, but it needs longer time to achieve it. For this scenario, we run 300,000 units of time and check the average populations over the last 1,000 units of time. If the mean population of the superior species falls between 0.001 and 0.6, we run another 100,000 units of time and continue to simulate 100,000 units of time until the superior is regard as extinct (mean population <0.001) or total simulation time reach 5,000,000 units of time.

Finally, the competitive outcome was determined over the last 1,000 units of time. A species is regarded as extinct if the average population over the last 1,000 units of time is below 0.001; and a system is regarded as stable if the average c.v. of surviving consumer populations is below 0.01.

**Simulation for invader fitness**

We evaluate fitness of the inferior by calculating its average growth rate when it starts to invade the C*_S_-R* system. In order to calculate the average growth rate, we simulated a model where the inferior has no effect on the resource, by the following processes:

First, let a sub-model with only the superior consumer and the resource (hereafter, *C_S_-R* system) reaches its long-term dynamics by simulating 1,500 units of time. Initial value of resource populations are at carrying capacities, and the superior consumer populations are at 0.001 in each patch.

Second, introduce very small populations of the inferior as the invader (10^-7^ for each patch) to the *C_S_-R* system.

Third, continue to simulate the model for 400 units of time. We record the average of total population size in the interval t=51~250 as A, and the average of total population size in the interval t=200~400 as B. The average growth rate was calculated as $\frac{\log_{e}\left(\frac{B}{A}\right)}{150}$.

**Reference**

1. Press WH (1988) Numerical recipes in C : the art of scientific computing. New York: Cambridge University Press.
