# Supplementary material for: Difference Inadaptive Dispersal Ability Can Promote Species Coexistence in Fluctuating Environments
Source: PLoS One. 2013 Feb 1;8(2):e55218. doi: 10.1371/journal.pone.0055218 (PMC3562337; doi:10.1371/journal.pone.0055218)
Supplement: Text S4 — Fitness differential of switching patches. (DOCX) [file pone.0055218.s011.docx]

**Text S4** **Fitness differential of switching patches**

We calculated the average fitness differential that the dispersers experienced when they change patches. At each time step, the “fitness differential of switching patches” ($\Delta F(t)$), was calculated as:

$$\Delta F(t)=\frac{C_{N,1}}{C_{N,1}+C_{N,2}}d_{outN,1}(f_{N,2}-f_{N,1})+\frac{C_{N,2}}{C_{N,1}+C_{N,2}}d_{outN,2}(f_{N,1}-f_{N,2}),$$

where *d*_out_*_N_*_,1_ and *d*_out_*_N_*_,2_ are emigration out of patch 1 and patch 2, and *f_N_*_,1_ and *f_N_*_,2_ are the fitness at patch 1 and patch 2, respectively. This value implies the density-weighted average of the change in the fitness through dispersal at each time *t*. We calculate the temporal average of$\Delta F(t)$ for *t* = 50~200 after invasion of the inferior. In order to avoid the effect of invader on the resource, we use a model where the inferior has no effect on the resource. The results of average fitness differential of switching patches are presented in supplementary Figure S1. However, this value may not always work as an adaptiveness measure because fitness at one patch may change after dispersers’ arrival in the non-equilibrium cases. In our model, the pattern of the fitness differential did not help to understand the consequences of competition shown in Figure 1. Nevertheless, the comparison between this fitness differential of switching patches and adaptiveness would allow us to better understand the mechanism of coexistence in general**.**
