# Supplementary material for: Difference Inadaptive Dispersal Ability Can Promote Species Coexistence in Fluctuating Environments
Source: PLoS One. 2013 Feb 1;8(2):e55218. doi: 10.1371/journal.pone.0055218 (PMC3562337; doi:10.1371/journal.pone.0055218)
Supplement: Text S5 — Detailed community dynamics in the scenario with random dispersal in environment 3. (DOCX) [file pone.0055218.s012.docx]

**Text S5** **Detailed community dynamics in the scenario with random dispersal in environment 3**

In the environment with high carrying capacity (environment 3), random dispersal basically promotes coexistence when the moving capacity of the superior is high and when the moving capacity of the inferior is low. In addition, other two outcomes were observed.

In the first case, the inferior could outcompete the superior in some cases when the moving capacity was high for the superior and moderate for the inferior. Figure S2 shows the community dynamics observed when the superior moving capacity is very high (*d*_max,_*_S_* = 100) and when the inferior is absent. This corresponds to the situation that the inferior faces when it invades. Populations of the superior were almost equal in both patches. The high population in patch 1 (supported by the high resource level) suppressed the resource in patch 2, which is thus kept zero. The inferior with very low moving capacity (e.g. *d*_max,_*_I_* = 0.01, Figure S3A) can invade this community and coexist with the superior because it takes advantage by staying in the more favorable patch. By staying in patch 1, the inferior reduced the resource level in patch1 and the population of the superior, thus the predation pressure of the superior in patch 2 was reduced, and resource level in patch 2 was not zero anymore (Fig. S3A). When the inferior moving capacity becomes higher (e.g., *d*_max,_*_I_* = 0.1, Figure S3 B), the inferior can still invade, but it cannot resolve the predation pressure in patch 2 enough to make it non-zero. The situation that one patch has no resource at all is especially harmful to the species with high moving capacity because the half of its population is not growing at all. This is why the inferior could eventually competitively exclude the superior. Moderate moving capacity of the inferior (e.g. *d*_max,_*_I_* = 1, Figure S3 C) would stabilize the resource dynamics, but it could still outcompete the superior, which is observed in the region I* in Figure 1C. When the moving capacity of the inferior becomes higher, its advantage gets weaker and cannot exclude the superior (e.g. *d*_max,_*_I_* = 1, Figure S3D), which is observed in the region Co^ in Figure 1C. The coexistence dynamics here is different from where the inferior moving capacity was very low (Figure S3A); with even higher moving capacity, the inferior cannot invade at all. Alternative states exist in a limited parameter region between S^ and I^ regions (Figure S5). As we shown in Figure 1C, the transition from S^ to I^ is abrupt when the inferior was simulated as invader (Figure S5A). However, when the superior consumer is simulated as the invader, coexistence with fluctuating population dynamics was also possible (Figure S5B).

In the second case, coexistence was realized when both species had low moving capacity (bottom-left corner of Figure 1C). In environment 3, random dispersal can also promote coexistence when both species had low moving capacity but the inferior moving capacity was higher. The community dynamics and the net movement of the inferior are shown in Figure S4. For most of time in the cyclic periods of fluctuations, direction of net movement of the inferior was toward the patch of lower fitness. However, the net movements were highest not long before the identity of the more suitable patch changes. In this way, the inferior had higher ratio of its population in patch 1 when there has the higher resource level, compared to the case with the superior species. To achieve this coexistence, the moving capacity of the inferior cannot be too high; otherwise, random dispersal would equalize the populations in the two patches.
