# Supplementary material for: Difference Inadaptive Dispersal Ability Can Promote Species Coexistence in Fluctuating Environments
Source: PLoS One. 2013 Feb 1;8(2):e55218. doi: 10.1371/journal.pone.0055218 (PMC3562337; doi:10.1371/journal.pone.0055218)
Supplement: Text S6 — Detailed community dynamics in the scenario with growth-dependent dispersal in environment 3. (DOCX) [file pone.0055218.s013.docx]

**Text S6** **Detailed community dynamics in the scenario with growth-dependent dispersal in environment 3**

In the environment with high carrying capacity (environment 3), growth-dependent dispersal promotes coexistence under two conditions: (1) moving capacity was low for the superior and high for the inferior consumer (top-left corner in Figure 1I), and (2) moving capacity was high for the superior and low for the inferior consumer (bottom-right corner in Figure 1I).

In the first case, coexistence is realized when moving capacity was low for the superior and high for the inferior consumer. One example (where *d*_max_*_,S_* =0.01, *d*_max_*_,I_* = 100) of community dynamics in this situation is provided in supplementary Figure S6. In this case, the resource levels were sometimes higher in patch 1 and sometimes higher in patch 2, and the inferior had more of it population stay in patch 1 when there had more resource, and had more of its population stay in patch 2 when there had more resource. The inferior takes more advantage by accumulating its population in the patch with higher resource level than the almost sedentary superior species. This advantage allows the inferior to coexist with the superior. This distribution was not mainly caused by the net movement toward the currently better patch. As can be seen in the lower panel of Figure S6, for the most time of one cyclic period of fluctuations, the net movement of the inferior was toward the patch only with lower fitness. However, the net movement toward patch 1 was highest not long before patch 1 became a better patch (patch of higher resource level), and the net movement toward patch 2 was higher not long before patch 2 became better. In this way, growth-dependent dispersal performed the resource-tracking behavior similar to fitness-dependent dispersal.

In the second case, coexistence is realized when moving capacity was high for the superior and low for the inferior consumer. One example (where *d*_max_*_,S_* = 100, *d*_max_*_,I_* = 0.01) of community dynamics in this situation is provided in supplementary Figure S7. In this case, resource levels were sometimes higher in patch 1, and sometimes almost equal in both patches (the middle panel in Figure S7). However, the fast-moving superior had their net movement toward patch 2 (the lower panel in Figure S7), resulting in lower ratio of its population staying in patch 1 than that of the almost sedentary inferior. Thus, the predation pressure in patch 1 was relaxed, and the almost sedentary inferior could invade and coexist with the superior.
